# *Cis*-epistasis at the *LPA* locus and risk of cardiovascular diseases

**DOI:** 10.1093/cvr/cvab136

**Published:** 2021-04-20

**Authors:** Lingyao Zeng, Sylvain Moser, Nazanin Mirza-Schreiber, Claudia Lamina, Stefan Coassin, Christopher P Nelson, Tarmo Annilo, Oscar Franzén, Marcus E Kleber, Salome Mack, Till F M Andlauer, Beibei Jiang, Barbara Stiller, Ling Li, Christina Willenborg, Matthias Munz, Thorsten Kessler, Adnan Kastrati, Karl-Ludwig Laugwitz, Jeanette Erdmann, Susanne Moebus, Markus M Nöthen, Annette Peters, Konstantin Strauch, Martina Müller-Nurasyid, Christian Gieger, Thomas Meitinger, Elisabeth Steinhagen-Thiessen, Winfried März, Andres Metspalu, Johan L M Björkegren, Nilesh J Samani, Florian Kronenberg, Bertram Müller-Myhsok, Heribert Schunkert

**Affiliations:** 1 Deutsches Herzzentrum München, Klinik für Herz- und Kreislauferkrankungen, Technische Universität München, 80636 Munich, Germany; 2 Department of Translational Research in Psychiatry, Max Planck Institute of Psychiatry, 80804 Munich, Germany; 3 International Max Planck Research School for Translational Psychiatry (IMPRS-TP), Munich 80804, Germany; 4 Institute of Neurogenomics, Helmholtz Zentrum München, 85764 Neuherberg, Germany; 5 Institute of Genetic Epidemiology, Department of Genetics and Pharmacology, Medical University of Innsbruck, Innsbruck 6020, Austria; 6 Department of Cardiovascular Sciences, University of Leicester, BHF Cardiovascular Research Centre, Glenfield Hospital, Groby Rd, Leicester LE3 9QP, UK; 7 NIHR Leicester Biomedical Research Centre, Glenfield Hospital, Leicester LE3 9QP, UK; 8 Estonian Genome Center, Institute of Genomics, University of Tartu, 51010 Tartu, Estonia; 9 Department of Genetics and Genomic Sciences and Institute for Genomics and Multiscale Biology, Icahn School of Medicine at Mount Sinai, One Gustave L. Levy Place, New York, NY 10029, USA; 10 Integrated Cardio Metabolic Centre, Karolinska Institutet, Huddinge, 14186 Stockholm, Sweden; 11 Medizinische Klinik V (Nephrologie, Hypertensiologie, Rheumatologie, Endokrinologie, Diabetologie), Medizinische Fakultät Mannheim der Universität Heidelberg, 69120 Heidelberg, Germany; 12 Department of Neurology, Klinikum rechts der Isar, School of Medicine, Technical University of Munich, 81675 Munich, Germany; 13 Institute for Cardiogenetics and University Heart Center Luebeck, University of Lübeck, 23562 Lübeck, Germany; 14 Deutsches Zentrum für Herz- und Kreislauf-Forschung (DZHK), Partner Site Hamburg/Lübeck/Kiel, 23562 Lübeck, Germany; 15 Charité – University Medicine Berlin, Corporate Member of Freie Universität Berlin, Humboldt-Universität zu Berlin, and Berlin Institute of Health, Institute for Dental and Craniofacial Sciences, Department of Periodontology and Synoptic Dentistry, 14197 Berlin, Germany; 16 Deutsches Zentrum für Herz- und Kreislauf-Forschung (DZHK), Partner Site Munich Heart Alliance, 80636 Munich, Germany; 17 Medizinische Klinik, Klinikum rechts der Isar, Technische Universität München, 81675 Munich, Germany; 18 Institute for Medical Informatics, Biometry and Epidemiology, University Hospital Essen, 45147 Essen, Germany; 19 Centre for Urbane Epidemiology, University Hospital Essen, 45147 Essen, Germany; 20 Institute of Human Genetics, University of Bonn School of Medicine & University Hospital Bonn, 53012 Bonn, Germany; 21 Institute of Genetic Epidemiology, Helmholtz Zentrum München, German Research Center for Environmental Health, 85764 Neuherberg, Germany; 22 IBE, Faculty of Medicine, LMU Munich, 81377 Munich, Germany; 23 Institute of Medical Biostatistics, Epidemiology and Informatics (IMBEI), University Medical Center, Johannes Gutenberg University, 55101 Mainz, Germany; 24 Department of Internal Medicine I (Cardiology), Hospital of the Ludwig-Maximilians-University (LMU) Munich, 81377 Munich, Germany; 25 Institute of Epidemiology II, Helmholtz Zentrum München, 85764 Neuherberg, Germany; 26 Institute of Human Genetics, Helmholtz Zentrum München, 85764 Neuherberg, Germany; 27 Center for Internal Medicine with Gastroenterology and Nephrology, Lipid Clinic, Charité, 13353 Berlin, Germany; 28 Synlab Akademie, Synlab Holding Deutschland GmbH, Mannheim und Augsburg, 86156 Augsburg, Germany; 29 Institute of Molecular and Cell Biology, University of Tartu, 51010 Tartu, Estonia; 30 Munich Cluster of Systems Biology, SyNergy, 81377 Munich, Germany; 31 Department of Health Data Science, University of Liverpool, Liverpool L69 3BX, UK

**Keywords:** Statistical genetics, Epistasis, Coronary artery diseases, LPA

## Abstract

**Aims:**

Coronary artery disease (CAD) has a strong genetic predisposition. However, despite substantial discoveries made by genome-wide association studies (GWAS), a large proportion of heritability awaits identification. Non-additive genetic effects might be responsible for part of the unaccounted genetic variance. Here, we attempted a proof-of-concept study to identify non-additive genetic effects, namely epistatic interactions, associated with CAD.

**Methods and results:**

We tested for epistatic interactions in 10 CAD case–control studies and UK Biobank with focus on 8068 SNPs at 56 loci with known associations with CAD risk. We identified a SNP pair located in *cis* at the *LPA* locus, rs1800769 and rs9458001, to be jointly associated with risk for CAD [odds ratio (OR) = 1.37, *P* = 1.07 × 10^−11^], peripheral arterial disease (OR = 1.22, *P* = 2.32 × 10^−4^), aortic stenosis (OR = 1.47, *P* = 6.95 × 10^−7^), hepatic lipoprotein(a) (Lp(a)) transcript levels (beta = 0.39, *P* = 1.41 × 10^−8^), and Lp(a) serum levels (beta = 0.58, *P* = 8.7 × 10^−32^), while individual SNPs displayed no association. Further exploration of the *LPA* locus revealed a strong dependency of these associations on a rare variant, rs140570886, that was previously associated with Lp(a) levels. We confirmed increased CAD risk for heterozygous (relative OR = 1.46, *P* = 9.97 × 10^−32^) and individuals homozygous for the minor allele (relative OR = 1.77, *P* = 0.09) of rs140570886. Using forward model selection, we also show that epistatic interactions between rs140570886, rs9458001, and rs1800769 modulate the effects of the rs140570886 risk allele.

**Conclusions:**

These results demonstrate the feasibility of a large-scale knowledge-based epistasis scan and provide rare evidence of an epistatic interaction in a complex human disease. We were directed to a variant (rs140570886) influencing risk through additive genetic as well as epistatic effects. In summary, this study provides deeper insights into the genetic architecture of a locus important for cardiovascular diseases.

## Graphical Abstract

**Figure:**
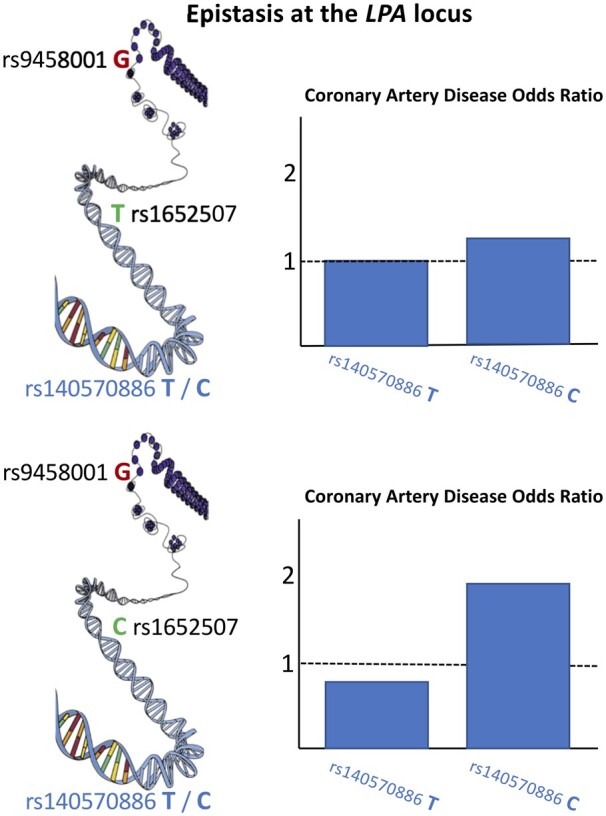

## 1 Introduction

Coronary artery disease (CAD) is one of the largest contributors to morbidity and mortality worldwide.^1^ A fundamental aspect of CAD is its complex and multi-factorial aetiology, which includes numerous environmental risk factors, such as obesity and smoking,^2^ as well as a strong genetic predisposition. Overall, the genetic variance is estimated to explain 40–50% of the variability in disease manifestation.^3^

A decade of genome-wide association studies (GWAS) shed light on the genetic architecture of the disease, discovering 163 genetic loci associated with CAD risk.^4^^,^^5^ About a quarter of CAD heritability can be explained by additive effects of these and other common genetic variants.^4^^,^^5^ More complex models involving gene regulatory networks^6^ may help to better explain the heritability of the disease. In addition, at some of these loci, multiple independent signals were described, showing intra-locus allelic heterogeneity.^7^ Until now, non-additive genetic effects, such as epistatic interactions, are largely neglected for explaining the heritability of CAD. However, epistasis has been postulated by some to account for part of this ‘missing heritability’^8^ and has also been found to act alongside additive effects to influence complex phenotypes.^9^^,^^10^

Epistatic interactions have profound effects in bacteria^11^ as well as in other higher model organisms^12^ and have been shown to regulate some quantitative traits in humans.^13^ However, evidence of epistasis in human genetics remains very scarce, because individual-level data with large sample sizes are required for epistasis studies. Moreover, the combinatorial nature of epistasis makes hypothesis-free genome-wide interaction analyses (GWIAs) computationally demanding and plagued with a high multiple testing burden. Finally, associations based on interactions appear to suffer from a low replication rate,^14^ and genetic interactions are sometimes difficult to disentangle from the tagging of haplotypes.^15^ Indeed, a non-causal combination of alleles at multiple SNPs co-inherited with a rare causal variant could act as a tag for this variant.

To face the computational complexities in search for interacting loci affecting risk for CAD, we conducted a two-stage statistical scanning procedure for epistasis using a GPU-accelerated software^16^ on individual-level data from several GWAS on CAD. The scan was based on susceptibility regions defined around the top 56 known CAD loci, thereby limiting the multiple testing correction burden while maximizing the likelihood to discover biologically relevant interactions.

## 2 Methods

### 2.1 Cohorts

#### 2.1.1 CAD case–control studies

Individual-level genotypes were obtained from 10 CAD case–control studies. From Germany: the German Myocardial Infarction Family Studies (GerMIFS) I,^17^ II,^18^ III (KORA),^19^ IV,^20^ V,^21^ VI^22^; the LUdwigshafen RIsk and Cardiovascular Health Study (LURIC)^23^; from Germany, England, and France: Cardiogenics; from England: Wellcome Trust Case Control Consortium (WTCCC)^24^^,^^25^; from France, Italy, Germany, and the USA: Myocardial Infarction Genetics Consortium (MIGen).^25^^,^^26^ Data from the WTCCC were obtained via the Leducq network ‘CADgenomics’ (https://www.fondationleducq.org/network/understanding-coronary-artery-disease-genes/). MIGen data were obtained via the database of Genotypes And Phenotypes (dbGaP; project ID #49717-3).^27^ The genotype processing procedures including QC and imputation are provided in Supplementary material online, *Methods*. The final sample sizes for each study after QC are listed in Supplementary material online, *Table S1*. All participants were of European origin and gave prior written informed consent, which specifically addressed that the materials will be used for genetic studies. All studies obtained institutional review board approval from their local Ethical Committees and were performed in accordance with the 1964 Helsinki Declaration and its later amendments. Ascertainment and assessment methods for CAD of each study are provided in the corresponding publications.

#### 2.1.2 UK Biobank

The UK Biobank (UKBB) project (http://www.ukbiobank.ac.uk) is a large prospective cohort study of ∼500 000 individuals from across the UK, aged 40–69 years at recruitment.^28^ In the present study, CAD cases were defined using the ‘SOFT’ and ‘HARD’ criteria,^22^ i.e., as individuals with fatal or nonfatal myocardial infarction (MI), percutaneous transluminal coronary angioplasty (PTCA), coronary artery bypass grafting (CABG), chronic ischaemic heart disease (IHD), and angina. Peripheral arterial disease (PAD) cases were defined as self-reported history of PAD, leg claudication/intermittent claudication or either hospitalization or death due to ICD9-443.9, ICD9-444, ICD10-I73.9, or ICD10-I74. Aortic valve stenosis cases were defined as a self-reported history of aortic stenosis or either hospitalization or death due to ICD9-424.1 or ICD10-I35.0. The post-imputation sample quality control (QC) performed in the UKBB dataset is detailed in the Supplementary material online, *Methods*. UKBB data were accessed under the approval of UKBB within project 9922. The study was conducted following the principles of the declaration of Helsinki and all participants gave prior written informed consent.

#### 2.1.3 KORA F3/F4 studies and STARNET-Study

Individual-level genotypes were obtained from population studies from Augsburg, Germany:^29^ KORA F3 and KORA F4^30^^,^^31^ along with lipid measurements including total lipoprotein(a) (Lp(a)) levels and the number of Kringle repeats of the Lp(a) protein. RNAseq data were generated from liver tissue of 522 CABG CAD patients from the Stockholm-Tartu Reverse Network Engineering Task (STARNET) study.^32^ These studies obtained institutional review board approval from their local Ethical Committees and were performed in accordance with the 1964 Helsinki Declaration and its later amendments. All participants gave prior written informed consent. Further information about these studies is provided in the Supplementary material online, *Methods*.

### 2.2 Epistasis scan

#### 2.2.1 Broad sense CAD susceptibility region

We focused our analysis on 56 loci with previous evidence from GWAS on CAD^20^^,^^25^ (Supplementary material online, *Table S14*) in order to restrict the number of variants for testing of statistical epistasis. Our aim was to enhance computation time and the likelihood of true positive findings by easing the multiple testing correction burden. CAD susceptibility regions were defined as ±500 kb around each of the 56 lead SNPs.^20^^,^^25^ This window size was chosen to capture the loci as completely as possible while minimizing the computational burden: the variance explained by the lead SNPs accounted for only 46% of the variance explained when including their flanking ±500 kb regions (Supplementary material online, *Figure S2* and *Methods*). We then pruned the variants in each region to 8,068 SNPs with pairwise *r*^2^ < 0.5 located in the broad CAD susceptibility regions.

#### 2.2.2 Statistical interaction analysis

**Figure 1:**
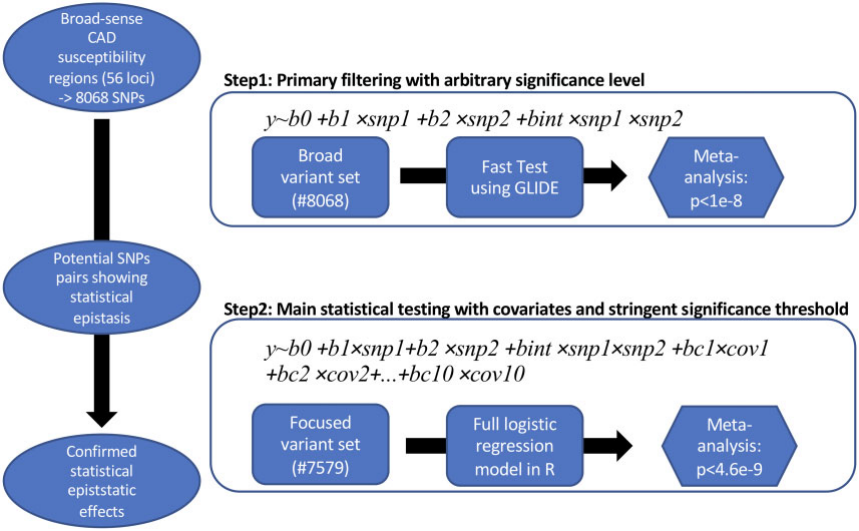
Scheme of the two-stage statistical interaction scanning procedure. Step 1 aimed at the fast identification of potential significant interaction terms using the GLIDE GPU computation tool. For each pair of LD-independent SNPs in the susceptibility regions (*N* = 8068 SNPs), we fitted a linear model with the additive and interaction effect of the two SNPs in each of the 10 CAD studies separately. The 10 *P*-values were then meta-analysed. A loose and arbitrary defined significance level (*P *<* *1e–8) was applied with the assumption that if there exists true epistasis between two lead SNPs, loose signals should be detectable between the SNPs within the corresponding LD block. Step 2 aimed at validating the results of the first step using logistic regression model including the first 10 multi-dimensional scaling (MDS) components of the genetic relationship matrix to correct for population structure. Step 2 also allowed the fine-mapping of candidate SNP pairs by screening for the strongest signal among all the SNPs within the LD blocks forwarded from Step 1. In this second step, we applied a stringent significance threshold of 4.6 × 10^−9,^ calculated as a Bonferroni correction (0.05/(*n*_SNP_indep_ × (*n*_SNP_indep_ − 1)/2) = 4.6178e−9) on the number of LD-independent SNPs resulting from Step 1 (*n*_SNP_indep_ = 4654).

We used the general framework for detecting statistical epistasis in quantitative genetics as proposed by Hansen and Wagner^33^ on the pairwise epistasis between two loci (SNPs) and implemented a two-stage statistical scanning procedure (*Figure 1*). The first step of the testing procedure consisted in a loose but fast statistical filtering using the GLIDE GPU computation tool.^16^ For each possible pair of SNPs, we fitted a linear model with the CAD phenotype as the dependent variable and the marginal effect of the two SNPs and their interaction term as predictors [Eq. (1)]. Each SNP’s genotype was encoded in four different models, dosage, dominant, recessive, and heterozygous with respect to the minor allele and all combinations of 2^4^ models were tested for each pair of SNPs.

(1) $$y∼b_{0}+b_{1}\times {\text{snp}}_{1}+b_{2}\times {\text{snp}}_{2}+b_{\text{int}}\times {\text{snp}}_{1}\times {\text{snp}}_{2}$$

A relatively loose and arbitrary significance level (*P *<* *1 × 10^−8^) was applied for primary filtering, with the assumption that if true epistasis existed between two SNPs, signals of moderate strength should be detectable between the SNPs within the corresponding linkage disequilibrium (LD) block. This threshold was defined with the aim to detect such pair in LD with the true epistasis signal and to forward a manageable number of pairs to the second step.

The second step included the fine-mapping of candidate SNP pairs to screen for the strongest signal among the SNPs in the same LD block. For this purpose, we used R to fit a logistic regression model, slower than the linear model used in step 1, but suited better for the binary CAD phenotype, and extended Eq. (1) to correct for population structure by adding the first 10 multi-dimensional scaling (MDS) components of the genetic relationship matrix [designated as MDS_1..10_ in Eq. (2) and following equations]. In this second step, we applied a stringent significance threshold of 4.6 × 10^−9,^ calculated as a Bonferroni correction (0.05/(*n*_SNP_indep_ × (n_SNP_indep_−1)/2) = 4.6178e−9) on the number of LD-independent SNPs resulting from Step 1 (*n*_SNP_indep_ = 4654). Each SNP pair was encoded in the genetic model displaying the highest significance in Step 1.

(2) $$y\sim b_{0}+b_{1}\times {\text{snp}}_{1}+b_{2}\times {\text{snp}}_{2}+b_{\text{int}}{\text{snp}}_{1}\times {\text{snp}}_{2}+{b_{c}}_{1}{\text{MDS}}_{1}+{b_{c}}_{2}\times {\text{MDS}}_{2}\mathrm{+...}+{b_{c}}_{10}\times {\text{MDS}}_{10}.$$

In the discovery phase, the same epistasis testing procedure was performed in each of the 10 CAD case–control studies separately. The models used genotype data imputed to the 1000 Genomes Phase 3 (1000GP3) reference panel. This regression analysis was followed by fixed-effects meta-analysis to estimate the overall effect size and standard error. The final epistasis pair of interest was then re-analysed in the same studies imputed using the Haplotype Reference Consortium (HRC) reference panel, to enable a more complete coverage of the region of interest in all 10 cohorts. Thereafter, this imputation based on the larger HRC reference was used for the remainder of the manuscript.

#### 2.2.3 Prioritizing candidate SNP pairs of epistasis of CAD

After the detection of SNP pairs showing statistically significant epistatic effects on the risk for CAD, we prioritized candidate pairs based on the following four criteria:

1. We retained only SNP pairs with a high replication potential [i.e. displaying statistical epistasis both significantly (*P *<* *4.6 × 10^−9^) and consistently (effect sizes pointing in the same direction) in at least eight of the 10 studies in the discovery data, based on both imputations].
2. LD between two target SNPs located on the same chromosome *r*^2^ < 0.2.
3. Weak interaction signals detectable between SNPs that show an LD *r*^2^ > 0.5 with any of the two interacting SNPs.
4. The effect of the interaction term is independent (i.e. *P*-value in conditional models <7.8 × 10^−6^) of any available third variant in conditional analyses.

### 2.3 Conditional analysis

The aim of the conditional analysis was to test whether the statistical epistasis effects were independent from a third SNP. To this end, we tested for the independence of the interaction term against the SNPs located within a ± 200kb window around the epistatic loci and any known CAD GWAS SNPs that survived the original QC procedure. This window size was chosen to capture all SNPs in significant LD with the pair of interest. Indeed, it has been shown that LD decay with physical distance and is close to 0 at 200 kb.^34^^,^^35^ For each of these SNPs, we used R to compute a likelihood ratio test (LRT) between a model including the additive effect of the two target SNPs and the additive effect of the conditioning SNP (all coded as minor allele dosages) [Eq. (3)] and a model including the interaction term in addition [Eq. (4)].

(3) $$y\sim b_{0}+b_{1}\times snp_{1}+b_{2}\times snp_{2}+b_{3}\times snp_{3}+{b_{c}}_{1}\times {\text{MDS}}_{1}+{b_{c}}_{2}\times {\text{MDS}}_{2}\text{+}...+{b_{c}}_{10}\times {\text{MDS}}_{10}$$

(4) $$y\sim b_{0}+b_{1}\times snp_{1}+b_{2}\times snp_{2}+b_{\mathrm{int}}\times snp_{1}\times snp_{2}+b_{3}\times snp_{3}+{b_{c}}_{1}\times {\text{MDS}}_{1}+{b_{c}}_{2}\times {\text{MDS}}_{2}\text{ +}...+{b_{c}}_{10}\times {\text{MDS}}_{10}$$

The interaction term was considered dependent on the conditioning SNP if the LRT did not reach a Bonferroni-corrected significance threshold defined on the total number of conditioning SNPs. This analysis was performed on a merged dataset of the 10 CAD studies. Here, the MDS components of the genetic relationship matrix used as covariates were re-calculated on the merged dataset.

### 2.4 Relative effect sizes and analyses of intermediate traits

Genotypic effect sizes for the different rs140570886 genotypes were computed by regression analysis in R using the dosage genetic model. Association analysis for the continuous intermediate traits Lp(a) protein levels, LPA mRNA levels, and KIV repeats were also performed using linear regression. Lp(a) proteins levels were highly skewed and Inverse Normal Transformation was applied prior association. The relative effect size for the three-SNP haplotypes was computed via haplotype estimation followed by fitting a generalized linear model with the R package *happassoc.* More detailed descriptions of these statistical procedures are provided in the Supplementary material online, *Methods*.

## 3 Results

### 3.1 Discovery of SNP pairs associated with CAD risk

**Figure 2:**
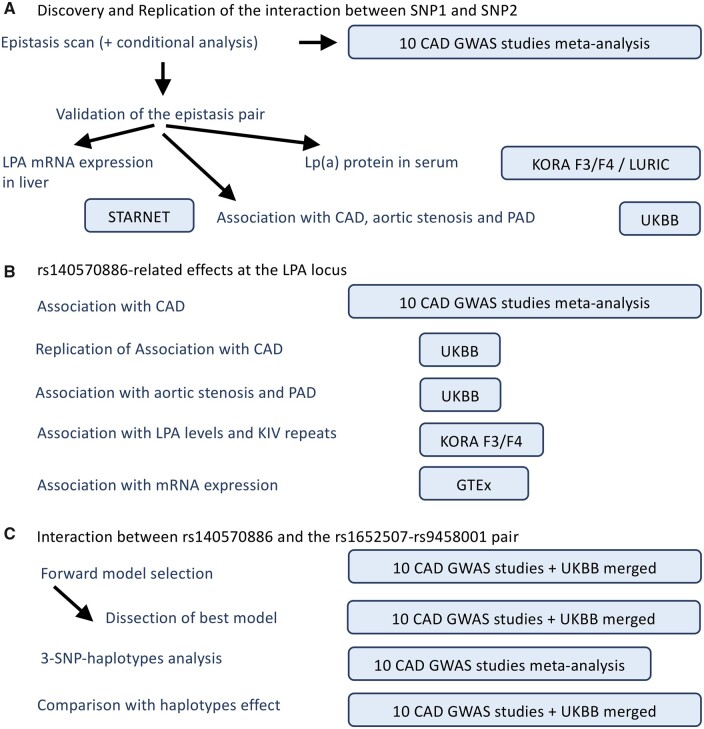
Analysis workflow and datasets. Schematic of the analysis workflow. (*A*) The epistasis scan was performed as a meta-analysis in the 10 CAD individual GWAS. Association of SNP1 (rs1800769) and SNP2 (rs9458001) interaction with CAD was replicated in the UK Biobank (UKBB). KORA F3/F4, LURIC, and STARNET were used for the association analysis of the interacting pair with proximal phenotypes. (*B*) The association of the additive effect of rs140570886 with CAD was assessed in a meta-analysis of the 10 CAD studies and replicated in the UKBB. KORA F3/F4 and GTEx were used for the association analysis of with rs140570886 proximal phenotypes. (*C*) The forward model selection, the dissection of the best model, and the comparison with the haplotypes effect were conducted on a merged dataset of the 10 CAD studies and the UKBB in order to achieve higher power. The 3-SNP haplotypes analysis on the other hand was carried out on the meta-analysis of the 10 CAD studies, because the algorithm used for fitting the Generalized Linear Model could not converge on the merged dataset which was too big.

We identified 56 previously known CAD risk loci from two previous GWAS^20^^,^^25^ (Supplementary material online, *Table S14*). For our study, we extracted 8068 LD-independent candidate variants within 500 kb of the respective lead SNPs. We observed that these extended regions explained more phenotypic variance than the respective lead SNPs alone (Supplementary material online, *Figure S2* and *Methods*). Testing for statistical interactions was carried out on all pairwise SNPs along with a two-step scheme (described in *Figure 1*) on imputed genotypes from 29 755 participants of 10 European CAD case–controls studies^17–22^^,^^24–26^ (*Figure 2*). Four SNP pairs displayed consistent (i.e. in at least 8 of 10 studies) and significant (i.e. *P* ≤ 4.618 × 10^−9^) effects and thus met our criteria as candidates for epistasis (Supplementary material online, *Table S2*). Among these four pairs, two (rs1800769 × rs9458001 and rs116632378× rs3823438) did replicate in the UKBB. The top SNP pair (rs1800769 × rs9458001) showed the strongest effect in a dosage–dosage model and was prioritized for further investigation (Supplementary material online, *Table S3*).

**Figure 3:**
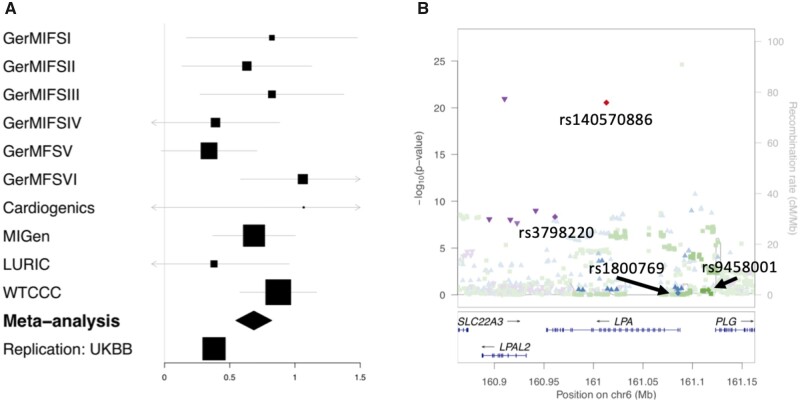
The common variant rs140570886, located in the *LPA* locus, increases CAD risk in a meta-analysis of 10 CAD studies and replicates in the UK Biobank. (*A*) Forest plot displaying the log odds ratio (OR) across 10 studies for rs140570886 as well as the fixed meta-analysis (*N* = 29 755) summary effect (shown as diamond) and the log OR in the replication dataset. The effect in the UK BioBank (UKBB, *N* = 312 312) is of the same sign and significant, therefore fulfilling the criteria for replication. (*B*) Manhattan plot showing the regional signal at the LPA locus taken from a recent genome-wide association study and indicating the variants in LD with rs140570886 (red), rs1800769 (blue), rs9458001 (green), and rs3798220 (purple).

Both rs1800769 and rs9458001 map to chromosome 6, close to the *LPA* locus (*Figure 3B*), and are not in LD with each other (*r*^2^ = 0.014, D = 0.535, *Table 1*). None of the SNPs were associated with CAD risk by itself in an additive model [*P* = 0.59, odds ratio (OR) = 0.99 for rs1800769[T]; *P* = 0.08, OR = 1.04 for rs9458001[A], Supplementary material online, *Table S2*]. However, the interaction term displayed a strong association (OR_int_ = 1.42, *P* = 1.75 × 10^−13^ for the rs1800769[T] × rs9458001[A] interaction term). In this case, as both SNP were encoded in the additive genetic model, the OR can be interpreted as the increase in likeliness to suffer from CAD associated with an increase of one unit in the product between the number of minor alleles at each of the interacting SNPs. The results were reproduced in the same dataset imputed with the HRC reference panel^36^ using rs1652507 (LD with rs1800769, *r*^2^ = 0.965, D’ = 0.991, *Table 1*) as a proxy for rs1800769 (OR = 0.98, *P* = 0.38 for rs1652507[C]; OR = 1.03, *P* = 0.1 for rs9458001[A], and OR_int_ = 1.36, *P* = 1.07 × 10^−11^ for the rs1652507[C] × rs9458001[A] interaction term, Supplementary material online, *Table S6*). Thus, the results were qualitatively independent of the imputation panel. This newer and denser imputation with this proxy variant was used for the remainder of the manuscript.

**Table 1:** Linkage **disequilibrium and minor allele frequency**

	rs3798220	rs140570886	rs1652507	rs1800769	rs9458001
rs3798220	**0.01932745**	0.703204	0.05355715	NA	0.0273448
rs140570886	0.703204	**0.01551685**	0.0759549	NA	0.0393634
rs1652507	0.05355715	0.0759549	**0.1591815**	*0.965*	0.0177401
rs1800769	NA	NA	*0.965*	NA	*0.014*
rs9458001	0.0273448	0.0393634	0.0177401	*0.014*	**0.2250345**

This table shows the pairwise *r*^2^ measure of Linkage Disequilibrium (LD) between the reported SNPs and their respective minor allele frequency (MAF) in bold on the diagonal. Values in Italic were computed in the European sub-samples of the 1000 Genomes Project using the LDmatrix tool (https://ldlink.nci.nih.gov/) as rs1800769 was absent from the HRC imputation panel. Other values were computed in each of the 10 CAD studies separately and averaged.

**Table 2:** ANOVA table reporting likelihood ratio test results for nested model in the model selection procedure

Model	Residuals. Df	Residuals deviance	Df	Deviance	*P*-value	***P*-value** **LRT model 2**
(1) CAD ∼ covariates	342 046	222 021	NA	NA	NA	NA
(2) CAD ∼ rs140570886 + covariates	342 045	221 804	1	217.04	4 × 10^−49^	NA
(3) CAD ∼ rs140570886 + rs9458005 + rs1652507 + covariates	342 043	221 770	2	33.57	5.1 × 10^−08^	5.1 × 10^−08^
(4) CAD ∼ rs140570886 + rs9458005 * rs1652507 + covariates	342 042	221 768	1	2.32	0.13	7.9 × 10^−08^
(5) CAD ∼ rs140570886 * rs9458005 * rs1652507 + covariates	342 039	221 755	3	13.42	0.004	6.5 × 10^−09^
(6) CAD ∼ rs140570886 * rs9458005 * rs1652507 +rs3798220+ covariates	342 038	221 753	1	1.93	0.16	8.2 × 10^−09^
(7) CAD ∼ rs140570886 * rs9458005 * rs1652507 * rs3798220+ covariates	342 031	221 746	7	6.22	0.51	8.2 × 10^−09^

The table displays the result of a series of successive likelihood ratio test between a nested model of increasing complexity performed on the merged dataset including the 10 CAD studies and the UK Biobank dataset. The first and second columns report the Residual Deviance and degrees of freedom of from each row’s model. The ‘Df’ and ‘Deviance’ columns respectively report the difference in degrees of freedom and deviance between each row’s model and the model from the previous row. The ‘*P*-value’ column reports the *P*-value of the likelihood ratio test between each row’s model and the previous one. The ‘*P*-value LRT model 2’ column reports the *P*-value of the likelihood ratio test between each model and the model containing only the additive effect of rs140570886. The * operator denotes factor crossing: a*b is interpreted as a + b+a × b and a*b*c as a + b+c+ a × b + a × c + b × c. The 10 multi-dimensional scaling components of the genetic variance and the study were included as covariates in every model. Tables showing the results of the same analysis with 3, 5, or 7 MDS components are provided in the Supplementary material online, *Tables S15–S17*.

**Table 3:** Model selection using AIC and Likelihood ratio test confirms epistatic interactions at the LPA locus

Model	Model name	AIC	Comparison M_SNPs	Comparison M_interact
No genetics	M_null	222 063.0	NA	NA
Haplotypes	M_haplo	221 813.6	NA	NA
SNPs	M_SNPs	221 818.3	NA	NA
SNPs + interactions	M_interact	221 810.6	0.0034	NA
Haplotypes + SNPs + interactions	M_full	221 814.4	0.0268	0.262

This table displays the Akaike Information Criterion (AIC) and results of likelihood ratio test for nested models of increasing complexity performed on the merged dataset including the 10 CAD studies and the UK Biobank dataset. The ‘Comparison M_SNPs’ and ‘Comparison M_interact’ columns respectively report the *P-*values of the likelihood ratio tests with the M_SNPs and M_interact models as null model. The 10 multi-dimensional scaling components of the genetic variance and were included as covariates in every model.

NA, non-applicable.

### 3.2 Replication and association with further traits

The UKBB dataset (controls/cases *n *= 285 520/26 792), used as an external replication sample (*Figure 2*), showed a consistent interaction effect of this SNP pair for CAD (OR_int_ = 1.15, *P* = 5.67 × 10^−10^ for the rs1652507 [C]×rs9458001[A] interaction term with the SNPs encoded in the dosage model, Supplementary material online, *Table S8*). Moreover, we found interaction effects in the same direction and with a comparable magnitude on peripheral vascular disease (controls/cases *n *= 475 059/4460, OR_int_ = 1.22, *P* = 2.32 × 10^−4^) and aortic valve stenosis (controls/cases *n *= 477 496/2,023, OR_int_ = 1.47, *P* = 6.95 × 10^−7^) (Supplementary material online, *Table S8*), conditions known to be affected by Lp(a) plasma levels.^37^^,^^38^

Next, we analysed the influence of the interaction term rs1800769×rs9458001 on circulating Lp(a) levels in a German population-based study (KORA F3/F4^30^^,^^31^*n *= 5953) (*Figure 2*). In addition to the association of each SNP separately, we identified a strong interaction effect of both SNPs on inverse-rank normal-transformed (INT) Lp(a) levels (beta = 0.58, *P* = 8.7 × 10^−32^, with the SNP encoded in the dosage model, Supplementary material online, *Table S6*). In the LURIC study, we replicated the significant statistical interaction for INT Lp(a) levels (beta = 0.56, *P* = 6.93 × 10^−16^) and found no other circulating factor displaying such effects (data not shown).

Finally, we extended our investigation to *LPA* mRNA expression in liver tissue (Section 2, STARNET study, *n *= 522) (*Figure 2*), where *LPA* is transcribed into Apo(a) and further assembled with an LDL-like particle into Lp(a). A significant interaction between the two SNPs was found (*P* = 1.4 × 10^-8^) and the effects on LPA mRNA expression correlated with the circulating Lp(a) levels measured in KORA F3/F4 for various genotype subgroups (Supplementary material online, *Table S6*), suggesting that differential gene expression activity underlies a large component of statistical interaction related to the two SNPs.

### 3.3 Rs140570886-related effects at the *LPA* locus

An inherent challenge in testing for epistasis of nearby SNPs, even if they are in very low LD, is to discriminate interacting SNPs from SNPs representing a specific haplotype. In order to explore the latter possibility, we assessed the interaction effect after conditioning for any known susceptibility SNPs for CAD (*n *= 158, Supplementary material online, *Table S4*) or any available SNP in the flanking ±200 kb region. The *LPA* region conditional analysis (see Section 2) did not yield any significant results (Supplementary material online, *Table S5*). However, studying GWAS lead SNPs (Supplementary material online, *Table S4*) uncovered that rs3798220 reduced the significance of the rs1652507×rs9458001 interaction term (increase from *P* = 1.07 × 10^−11^ to *P* = 2.08 × 10^−5^, likelihood ratio test).

In follow-up analyses, we also analysed the influence of the rare variant rs140570886 at the *LPA* locus, previously shown to be univariately associated with Lp(a) levels.^39^ This variant was not included in our primary analysis because its minor allele frequency was lower than our QC threshold (see Section 2), but was pointed out to us as requiring special attention. We therefore specifically investigated rs140570886 in the conditional analysis and observed a drastic decrease in the statistical support for the rs1652507×rs9458001 interaction term (from *P* = 8.95 × 10^−14^ to *P* = 0.022, likelihood ratio test). In order to test if these two SNPs (LD between rs140570886 and rs3798220: *r*^2^ = 0.808, D’ = 0.899) represented independent signals, we performed model selection using the likelihood ratio test. Adding rs3798220 to a model already containing rs140570886 did not improve the fit significantly (*P* = 0.49, likelihood ratio test). We therefore conclude that rs3798220 is not independent of rs140570886 and did not assess this SNP in further analyses.

We next investigated the additive effect of rs140570886 on CAD risk and found a significant association (OR = 1.98, *P* = 1.14 × 10^−21^, *Figure 3A*, Supplementary material online, *Table S10*). We replicated this association in the UKBB dataset (OR = 1.46, *P* = 2.77 × 10^−32^) (*Figure 3A*). Furthermore, In the UKBB, we found an association in the same direction and comparable magnitude for peripheral arterial disease (controls/cases *n *= 315 072/29 877, OR = 1.43, *P* = 7.83 × 10^−6^) and aortic valve stenosis (controls/cases *n *= 315 072/29 877, OR = 1.71, *P* = 1.25 × 10^−7^) (Supplementary material online, *Table S10*), both of which are manifestations of atherosclerosis in coronary arteries for which Lp(a) plasma levels affect risk.^37^^,^^38^

**Figure 4:**
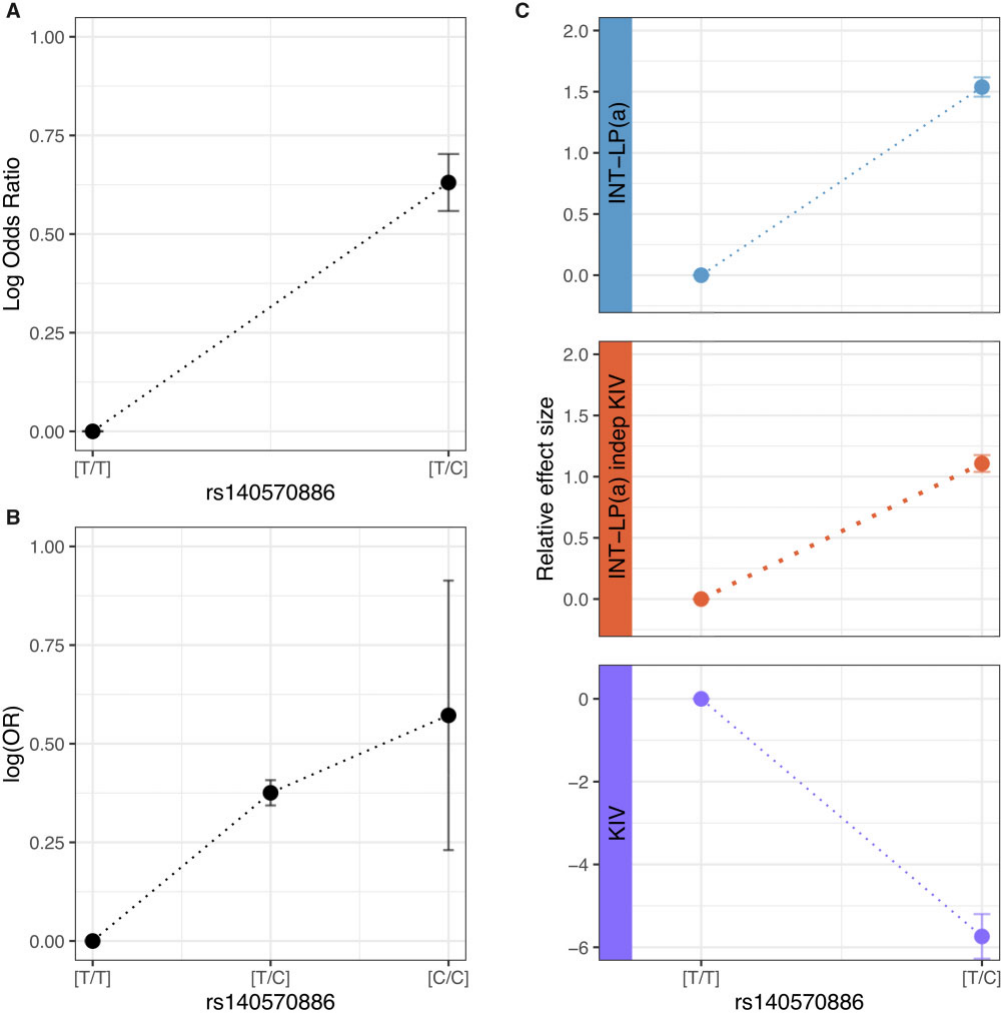
Genotype specific effect of rs140570886 on CAD risk and intermediate factors. (*A*) Genotypic log odds ratios (OR) (with reference to the genotype [T/T]) for the genotype subgroup [T/C] on CAD risk in the meta-analysis of ten CAD studies (*N* = 29 755). The OR for the minor allele homozygous genotype (C/C) is not displayed because of its low sample size and high standard error. Error bars represents the standard error of the log OR. (*B*) Genotypic OR (with reference to the genotype [T/T]) for the genotype subgroup [T/C] and [C/C] on CAD risk in the UK Biobank dataset (*N* = 312 312). Error bars represents the standard error of the log Odds Ratio. *C.* Relative effect size (with reference to the genotype [T/T]) for the genotype subgroup T/C on intermediate factors, namely inverse normal transformed Lp(a) levels (blue), KIV size of the dominantly expressed apo(a) isoform (purple), and the inverse normal transformed Lp(a) levels independent of the KIV (orange) in the KORA F3/F4 studies (*N* = 5953). Relative effect size for the minor allele homozygous genotype (C/C) is not displayed, because not represented in the KORA studies. Error bars represent standard error of the effect sizes.

To assess the contribution of rs140570886 genotypes to disease risk beyond the additive model, we next computed genotypic ORs for heterozygous [T/C] and minor allele homozygous genotypes [C/C] compared to the major allele homozygous reference genotype [T/T]. The genotypic model has the advantage that it does not make any assumption on the underlying genetic model. In the meta-analysis of the 10 CAD studies, we observed an OR of 1.88 (*P* = 2.32 × 10^−18^) for the T/C heterozygous genotype (*Figure 4A,*Supplementary material online, *Table S10*). A reliable effect estimate could not be calculated for the minor allele homozygous genotype C/C, due to its low frequency. The result for the T/C genotype was replicated in UKBB (OR = 1.46, *P* = 9.97 × 10^−32^) where we observed a trend for a higher relative OR for CC-homozygous subjects, although this was non-significant, likely due to its low frequency (OR = 1.77, *P* = 0.09; *Figure 4B*). This increase of the genotypic OR with the number of minor alleles suggests that the additive genetic model is indeed likely correct for rs140570886. Coherent with this, the saturated genotypic model does not provide a better fit than the additive model (*P* = 0.12, likelihood ratio test). We also observed a strong association of rs140570886 with Lp(a) levels (beta = 1.54, *P* = 9.52 × 10^−82^). As was the case for CAD risk, analyses of genotypic models indicated a linear increase with the minor allele count and thus supported an additive model (*Figure 4C*).

Circulating Lp(a) levels are modulated by at least two independent mechanisms.^40^ First, they are inversely correlated with the number of Kringle IV type 2 repeats (KIV-2 CNV),^41^^,^^42^ with fewer KIV-2 CNV repeats associated with more Lp(a) release from liver cells.^43^ They account for about 18% of the variability in Lp(a) levels in Western Europeans.^44^ However, individuals with the same number of KIV-2 CNV repeats may still differ up to 200-fold with respect to their Lp(a) levels,^41^^,^^42^ suggesting transcriptional mechanisms. In the KORA cohorts, we observed an association of rs140570886 with the KIV-2 CNV, with heterozygous rs140570886 carriers having fewer KIV-2 CNV repeats (beta = −5.74, *P* = 3.55 × 10^−26^) (Supplementary material online, *Table S10* and *Methods*). However, rs140570886 was in minimal LD with the reported 61 KIV-2 CNV-representing variants and the three independent modifier variants that influence the relationship between KIV-2 CNV and Lp(a) cholesterol^44^ (data not shown). More importantly, the effect of rs140570886 on Lp(a) levels remained highly significant after adjustment for the KIV-2 CNV (beta = 1.11, *P* = 1.94 × 10^−57^) (*Figure 4C*, Supplementary material online, *Table S10*). This strongly suggests that the effect of rs140570886 on Lp(a) levels is independent of the KIV-2 CNV and might therefore be modulated by transcriptional regulation. In accordance with this hypothesis, we found rs140570886 to be part of a significant expression quantitative trait locus (eQTL) with *LPA* mRNA expression levels in liver tissue, where *LPA* is transcribed to Apo(a) and further assembled into Lp(a) (GTEx V8, normalized effect size = 0.98, *P* = 1.2 × 10^−7^).

### 3.4 Interaction between rs140570886 and the rs1652507-rs9458001 pair

Although it appeared that part of the rs1652507×rs9458001 interaction was due to tagging of an rs140570886-related effect, we wondered if epistasis could still be present. To investigate this possibility, we applied a likelihood ratio tests-based forward model selection procedure starting with only rs140570886 going up to a model including all main effects and interactions between the four SNPs, rs1652507, rs9458001, rs140570886, and rs3798220 (Supplementary material online, *Methods*). To increase statistical power, we here analysed the CAD studies and UKBB jointly. We observed a significant increase in model fit when rs1652507 and rs9458001 were added as predictors to the model already containing rs140570886 (*Table 2*). The addition of the rs1652507×rs9458001 interaction term to this second model did not improve the fit further, coherent with the observed drop in the significance when conditioning the model containing the interaction term on rs140570886. However, the model fit increased significantly and reached its best level when all two-way and three-way interactions were added to the model. The direct comparison of this two-and-three-way interaction model to the rs140570886-only model yielded a *P*-value of the same magnitude as the original *P*-value threshold used for the epistasis screening (*P* = 6.46 × 10^−9^). Using a type-III Sum of Squares ANOVA to dissect this final model, provided further insights into the importance of the different coefficients (Supplementary material online, *Table S12*): we observed in the two-and-three-way interactions model that the additive effect of rs140570886 became non-significant while the additive effect of rs1652507 reached significance. Moreover, albeit the originally discovered rs1652507 × rs9458001 interaction term became non-significant, we observed nominally significant interactions of both rs1652507 and rs9458001 with rs140570886. These results suggest that the additive effect of rs140570886 on CAD risk might actually be caused by more complicated patterns of *cis*-epistatic interactions.

**Figure 5:**
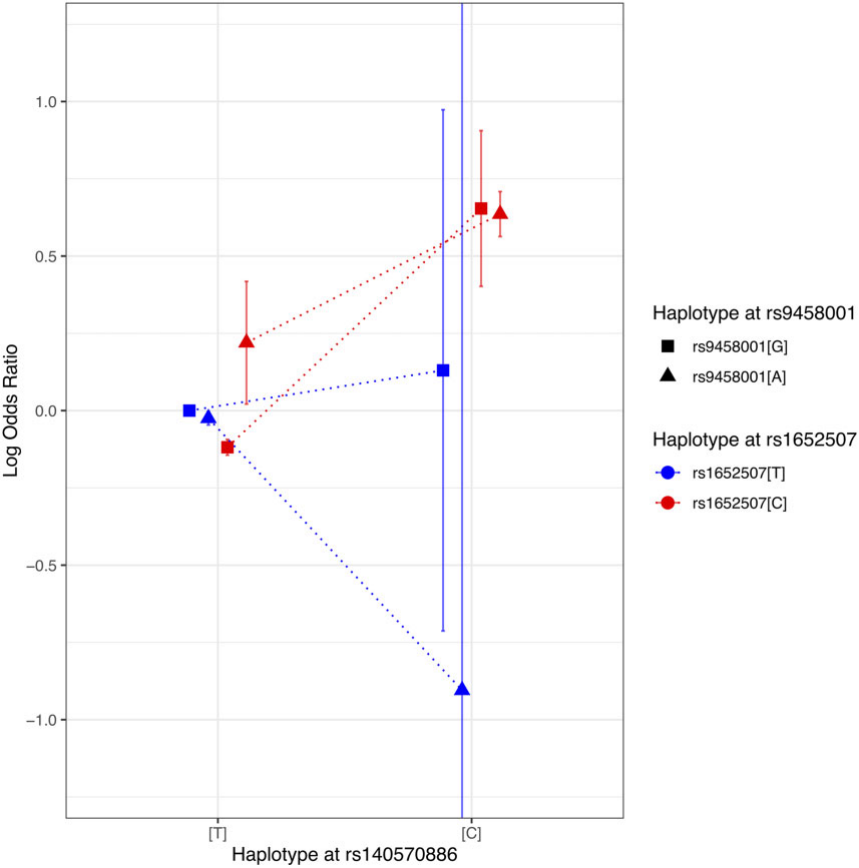
Relative effect of the rs140570886-rs1652507-rs9458001 3-SNP haplotypes on CAD risk. Relative odds ratio (OR; with reference to the most frequent TTG haplotypes) for the eight possible 3-SNP haplotypes on CAD risk. The red and blue colours represent the base at the rs1652507 SNP, the square and triangle shapes represent the base at the rs9458001 SNP and the position on the X-axis represent the base at the rs140570886 SNP. Together they indicate one of the eight possible 3-SNP haplotypes. The putative haplotypes were computed using the happasoc R package on a merged dataset of the 10 CAD studies (*N* = 29 755). Error bars represent the standard error of the log OR.

To better understand the genetics underlying this statistical model, we computed the relative OR for each of the eight possible haplotypes. It appeared that all haplotypes including the major T allele for rs140570886 showed similar ORs. Interestingly, we observed that the effect size varied profoundly across haplotypes containing the rs140570886 minor allele C, depending on the rs1652507 genotype (red vs. blue on *Figure 5*, Supplementary material online, *Table S13*). Moreover, for the haplotype rs140570886[C]—rs1652507[T], we observed that the ORs were much lower for the [A] as compared to the [G] allele at rs9458001, although the standard errors were large due to the low frequencies of rarer haplotypes (*Figure 5*, Supplementary material online, *Table S13*). These two observations reflect the marginally significant interaction coefficients between rs140570886 and rs1652507 and between rs140570886 and rs9458001 in the two- and three-way interaction model (Supplementary material online, *Table S12*).

Finally, in a further attempt to distinguish epistatic interactions involving these three SNPs from a haplotype effect, we compared different models containing SNPs encoded in the additive model and either haplotypes, interactions, or both, using the Akaike Information Criterion (AIC) (*Table 3*). The model including SNPs and their interactions but no haplotypes showed the best AIC. This result firstly confirms that interactions between the three SNPs improve model fit compared to an additive only model. Secondly, it suggests the presence of a real epistatic interaction between the three SNPs rather than an exclusive haplotype effect. The likelihood ratio test applied to the nested model confirmed this interpretation. Indeed, the most complex model, including SNPs, haplotypes, and interactions, did not provide a better fit than the one without the haplotypes (*P* = 0.26, likelihood ratio test), whereas adding interactions to the SNPs model improved the fit significantly (*P* = 0.0034, likelihood ratio test). Although we cannot exclude the involvement of other rare or non-typed variants and lack the statistical power necessary for these two interactions to reach the significance threshold pre-defined for the scan, these results demonstrate a complex genetic architecture involving non-additive and likely epistatic effects in the *LPA* region, underlying the regulation of Lp(a) expression and CAD risk.

## 4 Discussion

We report a two-stage testing procedure for epistatic interactions affecting CAD risk. Our analysis identified two SNPs at the *LPA* locus that individually had no effect but jointly displayed a strong statistical association with expression of *LPA* mRNA in liver, Lp(a) levels in serum, and with risk for CAD, peripheral arterial disease, and aortic stenosis. Further exploration of the locus revealed that parts of these associations were explained by tagging of a low-frequency variant (rs140570886), which, in parallel with our study, was found to be associated with Lp(a) levels.^39^ In addition, we detected a complex pattern of interactions between this variant and two other SNPs in the *LPA* region. Together, these findings firstly provide evidence of epistatic interaction in a complex human disease and provide deeper insights into the genetic architecture of an important locus for cardiovascular risk. At the same time, these data highlight the challenges in confirming epistatic interactions affecting disease risk in humans.

We focused our search for pairwise epistatic interactions on 8068 SNPs at 56 regions that had been found to be associated at genome-wide significance with CAD.^20^^,^^25^ Indeed, the selected window of LD-pruned SNPs contained two-fold more information on CAD heritability than the respective lead SNPs. Nevertheless, we found only four potentially interacting SNP pairs, which highlights the challenge to identify true epistasis modulating a human trait. The top-ranking interacting pair was located in *cis* at the *LPA* locus. Conditional analyses, aiming to determine the independence of the epistatic signal between rs1652507 and rs9458001 from other neighbouring SNPs, revealed a strong dependence on rs140570886. Thus, the seemingly strongest epistatic SNP pair tagged a rare genotype with profound effects on the phenotype. Further investigation of this variant showed its strong association with CAD and Lp(a) protein levels. rs140570886 has been previously associated with cardiovascular disease (CVD) using a new integrative framework named *FIODOR.*^45^ Our result, thus, using a well-established logistic regression model,^45^ confirmed the association of rs140570886 with diseases of the cardiovascular system. We, moreover, replicated the association of rs140570886 with Lp(a) levels reported by Mack *et al.*^39^ and identified data collections that indicate an association of rs140570886 with CAD.^7^ In addition, we report rs140570886 effects on Lp(a) levels to be independent of the KIV CNV repeats and to be a strong eQTL for *LPA* gene expression. Taken together, these findings support the hypothesis that rs140570886 mediates CAD risk through the Lp(a) levels via transcriptional regulation.

An important methodological point highlighted by this study is the importance of the conditional follow-up analyses in the investigation of epistatic interactions. Indeed, an inherent challenge in testing for epistasis of nearby SNPs, even if they are in very low LD, is to discriminate truly interacting SNPs from SNPs tagging a specific haplotype.^46^ Resolving the dependence structure at the epistatic locus, by conditioning the interaction effect on the neighbouring SNPs, allowed us to simultaneously identify a tagged rarer variant and to fine-map the epistatic interaction at the *LPA* locus.

The combinatorial nature of interactions has been a major hold-up in epistasis testing because it leads to an enormous search space and a high multiple testing correction burden.^15^^,^^47^ Methods to reduce this space can be divided into two categories: data-driven and knowledge-driven methods.^48^ We applied a data-driven approach in the present study, focusing on previously associated loci, for two reasons. First, variants already shown to be linked to the disease are likely to be functionally important. Second, if epistatic effects were detected among such variants, these effects would be more likely to affect the condition. Since regulatory variants might be located in the flanking region of the prioritized loci, we extended the search space to these regions. The discovery of four pairs of interacting SNPs using this filtering approach demonstrates its advantage over a hypothesis-free approach, in which these pairs would not have reached statistical significance due to having to correct for more tests.

The findings relative to the genetic architecture of the *LPA* locus reported in this study carry a special clinical relevance for CAD risk detection and treatment. Indeed, Lp(a) concentrations have been shown to be usable for CAD risk prediction. For example, the Copenhagen City Heart Study showed for individuals above the 95th percentiles of the Lp(a) concentration to have a 2.5-fold higher CAD risk compared to individuals in the lowest quartile.^37^ Although Lp(a) concentration measurement and isoform determination are sufficient assays to estimate CAD risk encoded at the *LPA* locus,^49^ polygenic risk scores might play an additional role in the assessment of CAD risk in the future.^5^ Indeed, with the rapid drop of genotyping cost, individual genotype data are becoming a basic component of biobanks and clinical settings.^50^ With this perspective, a better understanding of the genetic architecture of the *LPA* locus and the incorporation of non-additive genetic effects, such as those reported in this study, might enhance the predictive power of polygenic risk scores and help the development of individually tailored disease prevention,^51^ which, in the future, may involve a pharmacological Lp(a) reduction.^52^

While a single epistatic interaction as reported in this manuscript is very unlikely to improve risk prediction on its own compared to polygenic risk score based on millions of SNPs, numerous interactions—if identified—might do so. Indeed, several observations argue in this direction. First, simulations and analyses by others indicate that epistasis cannot be ruled out as an important factor.^10^ Particularly, results from the UK Biobank are compatible with an upper bound of epistasis explaining slightly more than half as much as additive variance, and a point estimate of epistasis explaining a quarter of the amount of variance explained by additively acting loci. A further extension of epistasis scans, to testing combinations of variants from disease susceptibility regions against the whole genome, or even to genome-wide scans with different, a-priori-defined functional, information-based filters, might discover new epistatic interactions,^53^ thereby, improving both our understanding of disease aetiology and possibly prediction models.

## Supplementary material

Supplementary material is available at *Cardiovascular Research* online.

## Supplementary Material

cvab136_Supplementary_Data

## References

[1] Naghavi M , Wang H, Lozano R, Davis A, Liang X, Zhou M, Vollset SE, Abbasoglu OA, Abdalla S, Abd-Allah F, Abdel AM, Abera SF, Aboyans V, Abraham B, Abraham JP, Abuabara KE, Abubakar I, Abu-Raddad LJ, Abu-Rmeileh NME, Achoki T, Adelekan A, Ademi Z, Adofo K, Adou AK, Adsuar JC, Ärnlov J, Agardh EE, Akena D, Khabouri MJ, Al Alasfoor D, Albittar M, Alegretti MA, Aleman AV, Alemu ZA, Alfonso-Cristancho R, Alhabib S, Ali MK, Ali R, Alla F, Lami F, Al Allebeck P, AlMazroa MA, Al-Shahi SR, Alsharif U, Alvarez E, Alviz-Guzman N, Amankwaa AA, Amare AT, Ameli O, Amini H, Ammar W, Anderson HR, Anderson BO, Antonio CAT, Anwari P, Apfel H, Argeseanu Cunningham S, Arsic AV, Artaman A, Asad MM, Asghar RJ, Assadi R, Atkins LS, Atkinson C, Badawi A, Bahit MC, Bakfalouni T, Balakrishnan K, Balalla S, Banerjee A, Barber RM, Barker-Collo SL, Barquera S, Barregard L, Barrero LH, Barrientos-Gutierrez T, Basu A, Basu S, Basulaiman MO, Beardsley J, Bedi N, Beghi E, Bekele T, Bell ML, Benjet C, Bennett DA, Bensenor IM, Benzian H, Bertozzi-Villa A, Beyene TJ, Bhala N, Bhalla A, Bhutta ZA, Bikbov B, Abdulhak A, Bin Biryukov S, Blore JD, Blyth FM, Bohensky MA, Borges G, Bose D, Boufous S, Bourne RR, Boyers LN, Brainin M, Brauer M, Brayne CEG, Brazinova A, Breitborde N, Brenner H, Briggs ADM, Brown JC, Brugha TS, Buckle GC, Bui LN, Bukhman G, Burch M, Campos Nonato IR, Carabin H, Cárdenas R, Carapetis J, Carpenter DO, Caso V, Castañeda-Orjuela CA, Castro RE, Catalá-López F, Cavalleri F, Chang JC, Charlson FC, Che X, Chen H, Chen Y, Chen JS, Chen Z, Chiang PPC, Chimed-Ochir O, Chowdhury R, Christensen H, Christophi CA, Chuang TW, Chugh SS, Cirillo M, Coates MM, Coffeng LE, Coggeshall MS, Cohen A, Colistro V, Colquhoun SM, Colomar M, Cooper LT, Cooper C, Coppola LM, Cortinovis M, Courville K, Cowie BC, Criqui MH, Crump JA, Cuevas-Nasu L, Costa Leite ID, Dabhadkar KC, Dandona L, Dandona R, Dansereau E, Dargan PI, Dayama A, La Cruz-Góngora VD, La VS, De Leo DD, Degenhardt L, Pozo-Cruz BD, Dellavalle RP, Deribe K, Jarlais DD, Dessalegn M, Veber GD, Dharmaratne SD, Dherani M, Diaz-Ortega JL, Diaz-Torne C, Dicker D, Ding EL, Dokova K, Dorsey ER, Driscoll TR, Duan L, Duber HC, Durrani AM, Ebel BE, Edmond KM, Ellenbogen RG, Elshrek Y, Ermakov SP, Erskine HE, Eshrati B, Esteghamati A, Estep K, Fürst T, Fahimi S, Fahrion AS, Faraon EJA, Farzadfar F, Fay DFJ, Feigl AB, Feigin VL, Felicio MM, Fereshtehnejad SM, Fernandes JG, Ferrari AJ, Fleming TD, Foigt N, Foreman K, Forouzanfar MH, Fowkes FGR, Fra Paleo U, Franklin RC, Futran ND, Gaffikin L, Gambashidze K, Gankpé FG, García-Guerra FA, Garcia AC, Geleijnse JM, Gessner BD, Gibney KB, Gillum RF, Gilmour S, Ginawi IAM, Giroud M, Glaser EL, Goenka S, Gomez DH, Gona P, Gonzalez-Medina D, Guinovart C, Gupta R, Gupta R, Gosselin RA, Gotay CC, Goto A, Gouda HN, Graetz N, Greenwell KF, Gugnani HC, Gunnell D, Gutiérrez RA, Haagsma J, Hafezi-Nejad N, Hagan H, Hagstromer M, Halasa YA, Hamadeh RR, Hamavid H, Hammami M, Hancock J, Hankey GJ, Hansen GM, Harb HL, Harewood H, Haro JM, Havmoeller R, Hay RJ, Hay SI, Hedayati MT, Heredia Pi IB, Heuton KR, Heydarpour P, Higashi H, Hijar M, Hoek HW, Hoffman HJ, Hornberger JC, Hosgood HD, Hossain M, Hotez PJ, Hoy DG, Hsairi M, Hu G, Huang JJ, Huffman MD, Hughes AJ, Husseini A, Huynh C, Iannarone M, Iburg KM, Idrisov BT, Ikeda N, Innos K, Inoue M, Islami F, Ismayilova S, Jacobsen KH, Jassal S, Jayaraman SP, Jensen PN, Jha V, Jiang G, Jiang Y, Jonas JB, Joseph J, Juel K, Kabagambe EK, Kan H, Karch A, Karimkhani C, Karthikeyan G, Kassebaum N, Kaul A, Kawakami N, Kazanjan K, Kazi DS, Kemp AH, Kengne AP, Keren A, Kereselidze M, Khader YS, Khalifa SEAH, Khan EA, Khan G, Khang YH, Kieling C, Kinfu Y, Kinge JM, Kim D, Kim S, Kivipelto M, Knibbs L, Knudsen AK, Kokubo Y, Kosen S, Kotagal M, Kravchenko MA, Krishnaswami S, Krueger H, Kuate DB, Kuipers EJ, Kucuk BB, Kulkarni C, Kulkarni VS, Kumar K, Kumar RB, Kwan GF, Kyu H, Lai T, Lakshmana BA, Lalloo R, Lallukka T, Lam H, Lan Q, Lansingh VC, Larson HJ, Larsson A, Lavados PM, Lawrynowicz AEB, Leasher JL, Lee JT, Leigh J, Leinsalu M, Leung R, Levitz C, Li B, Li Y, Li Y, Liddell C, Lim SS, Lima Gmf De Lind ML, Lipshultz SE, Liu S, Liu Y, Lloyd BK, Lofgren KT, Logroscino G, London SJ, Lortet-Tieulent J, Lotufo PA, Lucas RM, Lunevicius R, Lyons RA, Ma S, Machado VMP, MacIntyre MF, Mackay MT, MacLachlan JH, Magis-Rodriguez C, Mahdi AA, Majdan M, Malekzadeh R, Mangalam S, Mapoma CC, Marape M, Marcenes W, Margono C, Marks GB, Marzan MB, Masci JR, Mashal MT, Masiye F, Mason-Jones AJ, Matzopolous R, Mayosi BM, Mazorodze TT, McGrath JJ, McKay AC, McKee M, McLain A, Meaney PA, Mehndiratta MM, Mejia-Rodriguez F, Melaku YA, Meltzer M, Memish ZA, Mendoza W, Mensah GA, Meretoja A, Mhimbira FA, Miller TR, Mills EJ, Misganaw A, Mishra SK, Mock CN, Moffitt TE, Mohamed IN, Mohammad KA, Mokdad AH, Mola GL, Monasta L, Monis JDLC, Montañez HJ, Montico M, Montine TJ, Mooney MD, Moore AR, Moradi-Lakeh M, Moran AE, Mori R, Moschandreas J, Moturi WN, Moyer ML, Mozaffarian D, Mueller UO, Mukaigawara M, Mullany EC, Murray J, Mustapha A, Naghavi P, Naheed A, Naidoo KS, Naldi L, Nand D, Nangia V, Narayan KMV, Nash D, Nasher J, Nejjari C, Nelson RG, Neuhouser M, Neupane SP, Newcomb PA, Newman L, Newton CR, Ng M, Ngalesoni FN, Nguyen G, Nguyen NTT, Nisar MI, Nolte S, Norheim OF, Norman RE, Norrving B, Nyakarahuka L, Odell S, O’Donnell M, Ohkubo T, Ohno SL, Olusanya BO, Omer SB, Opio JN, Orisakwe OE, Ortblad KF, Ortiz A, Otayza MLK, Pain AW, Pandian JD, Panelo CI, Panniyammakal J, Papachristou C, Paternina CA, Patten SB, Patton GC, Paul VK, Pavlin B, Pearce N, Pellegrini CA, Pereira DM, Peresson SC, Perez-Padilla R, Perez-Ruiz FP, Perico N, Pervaiz A, Pesudovs K, Peterson CB, Petzold M, Phillips BK, Phillips DE, Phillips MR, Plass D, Piel FB, Poenaru D, Polinder S, Popova S, Poulton RG, Pourmalek F, Prabhakaran D, Qato D, Quezada AD, Quistberg DA, Rabito F, Rafay A, Rahimi K, Rahimi-Movaghar V, Rahman SUR, Raju M, Rakovac I, Rana SM, Refaat A, Remuzzi G, Ribeiro AL, Ricci S, Riccio PM, Richardson L, Richardus JH, Roberts B, Roberts DA, Robinson M, Roca A, Rodriguez A, Rojas-Rueda D, Ronfani L, Room R, Roth GA, Rothenbacher D, Rothstein DH, Rowley JTF, Roy N, Ruhago GM, Rushton L, Sambandam S, Søreide K, Saeedi MY, Saha S, Sahathevan R, Sahraian MA, Sahle BW, Salomon JA, Salvo D, Samonte GMJ, Sampson U, Sanabria JR, Sandar L, Santos IS, Satpathy M, Sawhney M, Saylan M, Scarborough P, Schöttker B, Schmidt JC, Schneider IJC, Schumacher AE, Schwebel DC, Scott JG, Sepanlou SG, Servan-Mori EE, Shackelford K, Shaheen A, Shahraz S, Shakh-Nazarova M, Shangguan S, She J, Sheikhbahaei S, Shepard DS, Shibuya K, Shinohara Y, Shishani K, Shiue I, Shivakoti R, Shrime MG, Sigfusdottir ID, Silberberg DH, Silva AP, Simard EP, Sindi S, Singh JA, Singh L, Sioson E, Skirbekk V, Sliwa K, So S, Soljak M, Soneji S, Soshnikov SS, Sposato LA, Sreeramareddy CT, Stanaway JD, Stathopoulou VK, Steenland K, Stein C, Steiner C, Stevens A, Stöckl H, Straif K, Stroumpoulis K, Sturua L, Sunguya BF, Swaminathan S, Swaroop M, Sykes BL, Tabb KM, Takahashi K, Talongwa RT, Tan F, Tanne D, Tanner M, Tavakkoli M, Ao B, Te Teixeira CM, Templin T, Tenkorang EY, Terkawi AS, Thomas BA, Thorne-Lyman AL, Thrift AG, Thurston GD, Tillmann T, Tirschwell DL, Tleyjeh IM, Tonelli M, Topouzis F, Towbin JA, Toyoshima H, Traebert J, Tran BX, Truelsen T, Trujillo U, Trillini M, Tsala Dimbuene Z, Tsilimbaris M, Tuzcu EM, Ubeda C, Uchendu US, Ukwaja KN, Undurraga EA, Vallely AJ, Vijver S Van De Gool CH, Van Varakin YY, Vasankari TJ, Vasconcelos AMN, Vavilala MS, Venketasubramanian N, Vijayakumar L, Villalpando S, Violante FS, Vlassov VV, Wagner GR, Waller SG, Wang JL, Wang L, Wang XR, Wang Y, Warouw TS, Weichenthal S, Weiderpass E, Weintraub RG, Wenzhi W, Werdecker A, Wessells KRR, Westerman R, Whiteford HA, Wilkinson JD, Williams TN, Woldeyohannes SM, Wolfe CDA, Wolock TM, Woolf AD, Wong JQ, Wright JL, Wulf S, Wurtz B, Xu G, Yang YC, Yano Y, Yatsuya H, Yip P, Yonemoto N, Yoon SJ, Younis M, Yu C, Yun JK, Zaki MES, Zamakhshary MF, Zeeb H, Zhang Y, Zhao Y, Zheng Y, Zhu J, Zhu S, Zonies D, Zou XN, Zunt JR, Vos T, Lopez AD, Murray CJL, Alcalá-Cerra G, Hu H, Karam N, Sabin N, Temesgen AM. Global, regional, and national age-sex specific all-cause and cause-specific mortality for 240 causes of death, 1990-2013: a systematic analysis for the Global Burden of Disease Study 2013. Lancet 2015;385:117–171. 25530442 10.1016/S0140-6736(14)61682-2 PMC4340604

[2] Yusuf S , Hawken S, Ôunpuu S, Dans T, Avezum A, Lanas F, McQueen M, Budaj A, Pais P, Varigos J, Lisheng L. Effect of potentially modifiable risk factors associated with myocardial infarction in 52 countries (the INTERHEART study): case-control study. Lancet 2004;364:937–952. 15364185 10.1016/S0140-6736(04)17018-9

[3] Myers RH , Kiely DK, Cupples LA, Kannel WB. Parental history is an independent risk factor for coronary artery disease: the Framingham Study. Am Heart J 1990;120:963–969. 2220549 10.1016/0002-8703(90)90216-k

[4] Erdmann J , Kessler T, Munoz VL, Schunkert H. A decade of genome-wide association studies for coronary artery disease: the challenges ahead. Cardiovasc Res 2018;114:1241–1257. 29617720 10.1093/cvr/cvy084

[5] Khera AV , Chaffin M, Aragam KG, Haas ME, Roselli C, Choi SH, Natarajan P, Lander ES, Lubitz SA, Ellinor PT, Kathiresan S. Genome-wide polygenic scores for common diseases identify individuals with risk equivalent to monogenic mutations. Nat Genet 2018;50:1219–1224. 30104762 10.1038/s41588-018-0183-z PMC6128408

[6] Zeng L , Talukdar HA, Koplev S, Giannarelli C, Ivert T, Gan LM, Ruusalepp A, Schadt EE, Kovacic JC, Lusis AJ, Michoel T, Schunkert H, Björkegren JLM. Contribution of gene regulatory networks to heritability of coronary artery disease. J Am Coll Cardiol 2019;73:2946–2957. 31196451 10.1016/j.jacc.2019.03.520 PMC6590059

[7] Van der Harst P , Verweij N. Identification of 64 novel genetic loci provides an expanded view on the genetic architecture of coronary artery disease. Circ Res 2018;122:433–443. 29212778 10.1161/CIRCRESAHA.117.312086 PMC5805277

[8] Zuk O , Hechter E, Sunyaev SR, Lander ES. The mystery of missing heritability: genetic interactions create phantom heritability. Proc Natl Acad Sci USA 2012;109:1193–1198. 22223662 10.1073/pnas.1119675109 PMC3268279

[9] Morgan MD , Pairo-Castineira E, Rawlik K, Canela-Xandri O, Rees J, Sims D, Tenesa A, Jackson IJ. Genome-wide study of hair colour in UK Biobank explains most of the SNP heritability. Nat Commun 2018;9:1–10. 30531825 10.1038/s41467-018-07691-z PMC6288091

[10] Hivert V , Sidorenko J, Rohart F, Goddard ME, Yang J, Wray NR, Yengo L, Visscher PM. Estimation of non-additive genetic variance in human complex traits from a large sample of unrelated individuals. Am J Hum Genet 2021;S0002-9297(21)00056-2. 10.1016/j.ajhg.2021.02.014 PMC8205999 33811805

[11] Costanzo M , VanderSluis B, Koch EN, Baryshnikova A, Pons C, Tan G, Wang W, Usaj M, Hanchard J, Lee SD, Pelechano V, Styles EB, Billmann M, Leeuwen JV, Dyk NV, Lin ZY, Kuzmin E, Nelson J, Piotrowski JS, Srikumar T, Bahr S, Chen Y, Deshpande R, Kurat CF, Li SC, Li Z, Usaj MM, Okada H, Pascoe N, Luis BJS, Sharifpoor S, Shuteriqi E, Simpkins SW, Snider J, Suresh HG, Tan Y, Zhu H, Malod-Dognin N, Janjic V, Przulj N, Troyanskaya OG, Stagljar I, Xia T, Ohya Y, Gingras AC, Raught B, Boutros M, Steinmetz LM, Moore CL, Rosebrock AP, Caudy AA, Myers CL, Andrews B, Boone C. A global genetic interaction network maps a wiring diagram of cellular function. Science (80-) 2016;353:aaf1420. 10.1126/science.aaf1420 PMC5661885 27708008

[12] Ganguly I , Anholt RRH, Kamdar KP, Mackay TFC, Chang S, Dilda CL, Kulkarni NH, Rollmann SM, Fanara J-J. The genetic architecture of odor-guided behavior in Drosophila: epistasis and the transcriptome. Nat Genet 2003;35:180–184. 12958599 10.1038/ng1240

[13] Mackay TFC. Epistasis and quantitative traits: using model organisms to study gene-gene interactions. Nat Rev Genet 2014;15:22–33. 24296533 10.1038/nrg3627 PMC3918431

[14] Murk W , Bracken MB, DeWan AT. Confronting the missing epistasis problem: on the reproducibility of gene–gene interactions. Hum Genet 2015;134:837–849. 25998948 10.1007/s00439-015-1564-3

[15] Ritchie MD , Steen KV. The search for gene-gene interactions in genome-wide association studies: challenges in abundance of methods, practical considerations, and biological interpretation. Ann Transl Med 2018;6:157–157. 29862246 10.21037/atm.2018.04.05 PMC5952010

[16] Kam-Thong T , Azencott C-A, Cayton L, Pütz B, Altmann A, Karbalai N, Sämann PG, Schölkopf B, Müller-Myhsok B, Borgwardt KM. GLIDE: GPU-based linear regression for detection of epistasis. Hum Hered 2012;73:220–236. 22965145 10.1159/000341885

[17] Nilesh S , Erdmann J, Hall AS, Mangino M, Mayer B, Dixon RJ, Meitinger T, Braund P, Wichmann H, Barrett JH, Tregouet A, Iles MM, Pahlke F, Pollard H, Lieb W, Cambien F, Fischer M, Blankenberg S, Balmforth A, König IR, Susanne S, Szymczak S, Tregouet D-A, Iles M, Pahlke F, Pollard H, Wolfgang L, Cambien F, Fischer M, Willem O, Stefan B, Balmforth AJ, Baessler A, Ball S, Strom TM, Brænne I, Gieger C, Deloukas P, Tobin M, Ziegler A, Thompson JR, Schunkert H. Genome wide association analysis of coronary artery disease. N Engl J Med 2007;357:443–453. 17634449 10.1056/NEJMoa072366 PMC2719290

[18] Erdmann J , Grosshennig A, Braund PS, König IR, Hengstenberg C, Hall AS, Linsel-Nitschke P, Kathiresan S, Wright B, Trégouët D-A, Cambien F, Bruse P, Aherrahrou Z, Wagner AK, Stark K, Schwartz SM, Salomaa V, Elosua R, Melander O, Voight BF, O'Donnell CJ, Peltonen L, Siscovick DS, Altshuler D, Merlini PA, Peyvandi F, Bernardinelli L, Ardissino D, Schillert A, Blankenberg S, Zeller T, Wild P, Schwarz DF, Tiret L, Perret C, Schreiber S, El Mokhtari NE, Schäfer A, März W, Renner W, Bugert P, Klüter H, Schrezenmeir J, Rubin D, Ball SG, Balmforth AJ, Wichmann H-E, Meitinger T, Fischer M, Meisinger C, Baumert J, Peters A, Ouwehand WH, Deloukas P, Thompson JR, Ziegler A, Samani NJ, Schunkert H; Cardiogenics Consortium. New susceptibility locus for coronary artery disease on chromosome 3q22.3. Nat Genet 2009;41:280–282. 19198612 10.1038/ng.307 PMC2695543

[19] Erdmann J , Willenborg C, Nahrstaedt J, Preuss M, Konig IR, Baumert J, Linsel-Nitschke P, Gieger C, Tennstedt S, Belcredi P, Aherrahrou Z, Klopp N, Loley C, Stark K, Hengstenberg C, Bruse P, Freyer J, Wagner AK, Medack A, Lieb W, Grosshennig A, Sager HB, Reinhardt A, Schafer A, Schreiber S, El Mokhtari NE, Raaz-Schrauder D, Illig T, Garlichs CD, Ekici AB, Reis A, Schrezenmeir J, Rubin D, Ziegler A, Wichmann H-E, Doering A, Meisinger C, Meitinger T, Peters A, Schunkert H. Genome-wide association study identifies a new locus for coronary artery disease on chromosome 10p11.23. Eur Heart J 2011;32:158–168. 21088011 10.1093/eurheartj/ehq405

[20] Nikpay M , Goel A, Won HH, Hall LM, Willenborg C, Kanoni S, Saleheen D, Kyriakou T, Nelson CP, CHopewell J, Webb TR, Zeng L, Dehghan A, Alver M, MArmasu S, Auro K, Bjonnes A, Chasman DI, Chen S, Ford I, Franceschini N, Gieger C, Grace C, Gustafsson S, Huang J, Hwang SJ, Kim YK, Kleber ME, Lau KW, Lu X, Lu Y, Lyytikäinen LP, Mihailov E, Morrison AC, Pervjakova N, Qu L, Rose LM, Salfati E, Saxena R, Scholz M, Smith AV, Tikkanen E, Uitterlinden A, Yang X, Zhang W, Zhao W, Andrade M, De Vries PS, De Zuydam NR, Van Anand SS, Bertram L, Beutner F, Dedoussis G, Frossard P, Gauguier D, Goodall AH, Gottesman O, Haber M, Han BG, Huang J, Jalilzadeh S, Kessler T, König IR, Lannfelt L, Lieb W, Lind L, MLindgren C, Lokki ML, Magnusson PK, Mallick NH, Mehra N, Meitinger T, Memon FUR, Morris AP, Nieminen MS, Pedersen NL, Peters A, Rallidis LS, Rasheed A, Samuel M, Shah SH, Sinisalo J, EStirrups K, Trompet S, Wang L, Zaman KS, Ardissino D, Boerwinkle E, Borecki IB, Bottinger EP, Buring JE, Chambers JC, Collins R, Cupples L, Danesh J, Demuth I, Elosua R, Epstein SE, Esko T, Feitosa MF, Franco OH, Franzosi MG, Granger CB, Gu D, Gudnason V, SHall A, Hamsten A, Harris TB, LHazen S, Hengstenberg C, Hofman A, Ingelsson E, Iribarren C, Jukema JW, Karhunen PJ, Kim BJ, Kooner JS, Kullo IJ, Lehtimäki T, Loos RJF, Melander O, Metspalu A, März W, Palmer CN, Perola M, Quertermous T, Rader DJ, Ridker PM, Ripatti S, Roberts R, Salomaa V, Sanghera DK, Schwartz SM, Seedorf U, Stewart AF, Stott DJ, Thiery J, Zalloua PA, O’Donnell CJ, Reilly MP, Assimes TL, Thompson JR, Erdmann J, Clarke R, Watkins H, Kathiresan S, McPherson R, Deloukas P, Schunkert H, Samani N, Farrall M. A comprehensive 1000 Genomes-based genome-wide association meta-analysis of coronary artery disease. Nat Genet 2015;47:1121–1130. 26343387 10.1038/ng.3396 PMC4589895

[21] Stitziel NO , Won HH, Morrison AC, Peloso GM, Do R, Lange LA, Fontanillas P, Gupta N, Duga S, Goel A, Farrall M, Saleheen D, Ferrario P, König I, Asselta R, Merlini PA, Marziliano N, Notarangelo MF, Schick U, Auer P, Assimes TL, Reilly M, Wilensky R, Rader DJ, Kees Hovingh G, Meitinger T, Kessler T, Kastrati A, Laugwitz KL, Siscovick D, Rotter JI, Hazen SL, Tracy R, Cresci S, Spertus J, Jackson R, Schwartz SM, Natarajan P, Crosby J, Muzny D, Ballantyne C, Rich SS, O’Donnell CJ, Abecasis G, Sunyaev S, Nickerson DA, Buring JE, Ridker PM, Chasman DI, Austin E, Ye Z, Kullo IJ, Weeke PE, Shaffer CM, Bastarache LA, Denny JC, Roden DM, Palmer C, Deloukas P, Lin DY, Tang ZZ, Erdmann J, Schunkert H, Danesh J, Marrugat J, Elosua R, Ardissino D, McPherson R, Watkins H, Reiner AP, Wilson JG, Altshuler D, Gibbs RA, Lander ES, Boerwinkle E, Gabriel S, Kathiresan S; Myocardial Infarction Genetics Consortium Investigators. Inactivating mutations in NPC1L1 and protection from coronary heart disease. N Engl J Med 2014;371:2072–2082. 25390462 10.1056/NEJMoa1405386 PMC4335708

[22] Nelson CP , Goel A, Butterworth AS, Kanoni S, Webb TR, Marouli E, Zeng L, Ntalla I, Lai FY, Hopewell JC, Giannakopoulou O, Jiang T, Hamby SE, Angelantonio E, Di Assimes TL, Bottinger EP, Chambers JC, Clarke R, Palmer CNA, Cubbon RM, Ellinor P, Ermel R, Evangelou E, Franks PW, Grace C, Gu D, Hingorani AD, Howson JMM, Ingelsson E, Kastrati A, Kessler T, Kyriakou T, Lehtimäki T, Lu X, Lu Y, März W, McPherson R, Metspalu A, Pujades-Rodriguez M, Ruusalepp A, Schadt EE, Schmidt AF, Sweeting MJ, Zalloua PA, Alghalayini K, Keavney BD, Kooner JS, Loos RJF, Patel RS, Rutter MK, Tomaszewski M, Tzoulaki I, Zeggini E, Erdmann J, Dedoussis G, Björkegren JLM, Schunkert H, Farrall M, Danesh J, Samani NJ, Watkins H, Deloukas P; EPIC-CVD Consortium. Association analyses based on false discovery rate implicate new loci for coronary artery disease. Nat Genet 2017;49:1385–1391. 28714975 10.1038/ng.3913

[23] Winkelmann BR , März W, Boehm BO, Zotz R, Hager J, Hellstern P, Senges J. Rationale and design of the LURIC study - a resource for functional genomics, pharmacogenomics and long-term prognosis of cardiovascular disease. Pharmacogenomics 2001;2:S1–21. 11258203 10.1517/14622416.2.1.S1

[24] Burton PR , Clayton DG, Cardon LR, Craddock N, Deloukas P, Duncanson A, Kwiatkowski DP, McCarthy MI, Ouwehand WH, Samani NJ, Todd JA, Donnelly P, Barrett JC, Davison D, Easton D, Evans D, Leung HT, Marchini JL, Morris AP, Spencer CCA, Tobin MD, Attwood AP, Boorman JP, Cant B, Everson U, Hussey JM, Jolley JD, Knight AS, Koch K, Meech E, Nutland S, Prowse CV, Stevens HE, Taylor NC, Walters GR, Walker NM, Watkins NA, Winzer T, Jones RW, McArdle WL, Ring SM, Strachan DP, Pembrey M, Breen G, St CD, Caesar S, Gordon-Smith K, Jones L, Fraser C, Green EK, Grozeva D, Hamshere ML, Holmans PA, Jones IR, Kirov G, Moskvina V, Nikolov I, O’Donovan MC, Owen MJ, Collier DA, Elkin A, Farmer A, Williamson R, McGuffin P, Young AH, Ferrier IN, Ball SG, Balmforth AJ, Barrett JH, Bishop DT, Iles MM, Maqbool A, Yuldasheva N, Hall AS, Braund PS, Dixon RJ, Mangino M, Stevens S, Thompson JR, Bredin F, Tremelling M, Parkes M, Drummond H, Lees CW, Nimmo ER, Satsangi J, Fisher SA, Forbes A, Lewis CM, Onnie CM, Prescott NJ, Sanderson J, Mathew CG, Barbour J, Mohiuddin MK, Todhunter CE, Mansfield JC, Ahmad T, Cummings FR, Jewell DP, Webster J, Brown MJ, Lathrop GM, Connell J, Dominiczak A, Braga Marcano CA, Burke B, Dobson R, Gungadoo J, Lee KL, Munroe PB, Newhouse SJ, Onipinla A, Wallace C, Xue M, Caulfield M, Farrall M, Barton A, Bruce IN, Donovan H, Eyre S, Gilbert PD, Hider SL, Hinks AM, John SL, Potter C, Silman AJ, Symmons DPM, Thomson W, Worthington J, Dunger DB, Widmer B, Frayling TM, Freathy RM, Lango H, Perry JRB, Shields BM, Weedon MN, Hattersley AT, Hitman GA, Walker M, Elliott KS, Groves CJ, Lindgren CM, Rayner NW, Timpson NJ, Zeggini E, Newport M, Sirugo G, Lyons E, Vannberg F, Hill AVS, Bradbury LA, Farrar C, Pointon JJ, Wordsworth P, Brown MA, Franklyn JA, Heward JM, Simmonds MJ, Gough SCL, Seal S, Stratton MR, Rahman N, Ban SM, Goris A, Sawcer SJ, Compston A, Conway D, Jallow M, Rockett KA, Bumpstead SJ, Chaney A, Downes K, Ghori MJR, Gwilliam R, Hunt SE, Inouye M, Keniry A, King E, McGinnis R, Potter S, Ravindrarajah R, Whittaker P, Widden C, Withers D, Cardin NJ, Ferreira T, Pereira-Gale J, Hallgrimsdóttir IB, Howie BN, Spencer CCA, Su Z, Teo YY, Vukcevic D, Bentley D, Compston A. Genome-wide association study of 14,000 cases of seven common diseases and 3,000 shared controls. Nature 2007;447:661–678. 17554300 10.1038/nature05911 PMC2719288

[25] Deloukas P , Kanoni S, Willenborg C, Farrall M, Assimes TL, Thompson JR, Ingelsson E, Saleheen D, Erdmann J, Goldstein BA, Stirrups K, König IR, Cazier J-B, Johansson Å, Hall AS, Lee J-Y, Willer CJ, Chambers JC, Esko T, Folkersen L, Goel A, Grundberg E, Havulinna AS, Ho WK, Hopewell JC, Eriksson N, Kleber ME, Kristiansson K, Lundmark P, Lyytikäinen L-P, Rafelt S, Shungin D, Strawbridge RJ, Thorleifsson G, Tikkanen E, Van Zuydam N, Voight BF, Waite LL, Zhang W, Ziegler A, Absher D, Altshuler D, Balmforth AJ, Barroso I, Braund PS, Burgdorf C, Claudi-Boehm S, Cox D, Dimitriou M, Do R, Doney ASF, Mokhtari NE, Eriksson P, Fischer K, Fontanillas P, Franco-Cereceda A, Gigante B, Groop L, Gustafsson S, Hager J, Hallmans G, Han B-G, Hunt SE, Kang HM, Illig T, Kessler T, Knowles JW, Kolovou G, Kuusisto J, Langenberg C, Langford C, Leander K, Lokki M-L, Lundmark A, McCarthy MI, Meisinger C, Melander O, Mihailov E, Maouche S, Morris AD, Müller-Nurasyid M, Nikus K, Peden JF, Rayner NW, Rasheed A, Rosinger S, Rubin D, Rumpf MP, Schäfer A, Sivananthan M, Song C, Stewart AFR, Tan S-T, Thorgeirsson G, Schoot CEVD, Wagner PJ, Wells GA, Wild PS, Yang T-P, Amouyel P, Arveiler D, Basart H, Boehnke M, Boerwinkle E, Brambilla P, Cambien F, Cupples AL, de Faire U, Dehghan A, Diemert P, Epstein SE, Evans A, Ferrario MM, Ferrières J, Gauguier D, Go AS, Goodall AH, Gudnason V, Hazen SL, Holm H, Iribarren C, Jang Y, Kähönen M, Kee F, Kim H-S, Klopp N, Koenig W, Kratzer W, Kuulasmaa K, Laakso M, Laaksonen R, Lee J-Y, Lind L, Ouwehand WH, Parish S, Park JE, Pedersen NL, Peters A, Quertermous T, Rader DJ, Salomaa V, Schadt E, Shah SH, Sinisalo J, Stark K, Stefansson K, Trégouët D-A, Virtamo J, Wallentin L, Wareham N, Zimmermann ME, Nieminen MS, Hengstenberg C, Sandhu MS, Pastinen T, Syvänen A-C, Hovingh GK, Dedoussis G, Franks PW, Lehtimäki T, Metspalu A, Zalloua PA, Siegbahn A, Schreiber S, Ripatti S, Blankenberg SS, Perola M, Clarke R, Boehm BO, O'Donnell C, Reilly MP, März W, Collins R, Kathiresan S, Hamsten A, Kooner JS, Thorsteinsdottir U, Danesh J, Palmer CNA, Roberts R, Watkins H, Schunkert H, Samani NJ; The CARDIoGRAMplusC4D Consortium. Large-scale association analysis identifies new risk loci for coronary artery disease. Nat Genet 2013;45:25–33. 23202125 10.1038/ng.2480 PMC3679547

[26] Kathiresan S , Voight BF, Purcell S, Musunuru K, Ardissino D, Mannucci PM, Anand S, Engert JC, Samani NJ, Schunkert H, Erdmann J, Reilly MP, Rader DJ, Morgan T, Spertus JA, Stoll M, Girelli D, McKeown PP, Patterson CC, Siscovick DS, O’Donnell CJ, Elosua R, Peltonen L, Salomaa V, Schwartz SM, Melander O, Altshuler D, Merlini PA, Berzuini C, Bernardinelli L, Peyvandi F, Tubaro M, Celli P, Ferrario M, Fetiveau R, Marziliano N, Casari G, Galli M, Ribichini F, Rossi M, Bernardi F, Zonzin P, Piazza A, Yee J, Friedlander Y, Marrugat J, Lucas G, Subirana I, Sala J, Ramos R, Meigs JB, Williams G, Nathan DM, MacRae CA, Havulinna AS, Berglund G, Hirschhorn JN, Asselta R, Duga S, Spreafico M, Daly MJ, Nemesh J, Korn JM, McCarroll SA, Surti A, Guiducci C, Gianniny L, Mirel D, Parkin M, Burtt N, Gabriel SB, Thompson JR, Braund PS, Wright BJ, Balmforth AJ, Ball SG, Hall AS, Linsel-Nitschke P, Lieb W, Ziegler A, König IR, Hengstenberg C, Fischer M, Stark K, Grosshennig A, Preuss M, Wichmann HE, Schreiber S, Ouwehand W, Deloukas P, Scholz M, Cambien F, Cardiogenics Li M, Chen Z, Wilensky R, Matthai W, Qasim A, Hakonarson HH, Devaney J, Burnett MS, Pichard AD, Kent KM, Satler L, Lindsay JM, Waksman R, Epstein SE, Scheffold T, Berger K, Huge A, Martinelli N, Olivieri O, Corrocher R, Hólm H, Thorleifsson G, Thorsteinsdottir U, Stefansson K, Do R, Xie C, Siscovick D. Genome-wide association of early-onset myocardial infarction with single nucleotide polymorphisms and copy number variants. Nat Genet 2009;41:334–341. 19198609 10.1038/ng.327 PMC2681011

[27] Tryka KA , Hao L, Sturcke A, Jin Y, Wang ZY, Ziyabari L, Lee M, Popova N, Sharopova N, Kimura M, Feolo M. NCBI’s database of genotypes and phenotypes: dbGaP. Nucleic Acids Res 2014;42:975–979. 10.1093/nar/gkt1211 PMC3965052 24297256

[28] Sudlow C , Gallacher J, Allen N, Beral V, Burton P, Danesh J, Downey P, Elliott P, Green J, Landray M, Liu B, Matthews P, Ong G, Pell J, Silman A, Young A, Sprosen T, Peakman T, Collins R. UK Biobank: an open access resource for identifying the causes of a wide range of complex diseases of middle and old age. PLoS Med 2015;12:e1001779. 25826379 10.1371/journal.pmed.1001779 PMC4380465

[29] Heid IM , Boes E, MüLler M, Kollerits B, Lamina C, Coassin S, Gieger C, Döring A, Klopp N, Frikke-Schmidt R, Tybjaerg-Hansen A, Brandstätter A, Luchner A, Meitinger T, Wichmann H-E, Kronenberg F. Genome-wide association analysis of high-density lipoprotein cholesterol in the population-based KORA study sheds new light on intergenic regions. Circ Cardiovasc Genet 2008;1:10–20. 20031538 10.1161/CIRCGENETICS.108.776708

[30] Wichmann HE , Gieger C, Illig T. KORA-gen - resource for population genetics, controls and a broad spectrum of disease phenotypes. Gesundheitswesen 2005;67:26–30. S30. 10.1055/s-2005-858226 16032514

[31] Holle R , Happich M, Löwel H, Wichmann HE. KORA - a research platform for population based health research. Gesundheitswesen 2005;67:19– 25. 10.1055/s-2005-858235 16032513

[32] Franzen O , Ermel R, Cohain A, Akers NK, Di Narzo A, Talukdar HA, Foroughi-Asl H, Giambartolomei C, Fullard JF, Sukhavasi K, Koks S, Gan L-M, Giannarelli C, Kovacic JC, Betsholtz C, Losic B, Michoel T, Hao K, Roussos P, Skogsberg J, Ruusalepp A, Schadt EE, Bjorkegren JLM. Cardiometabolic risk loci share downstream cis- and trans-gene regulation across tissues and diseases. Science *(80-)* 2016;353:827–830. 27540175 10.1126/science.aad6970 PMC5534139

[33] Hansen TF , Wagner GP. Modeling genetic architecture: a multilinear theory of gene interaction. Theor Popul Biol 2001;59:61–86. 11243929 10.1006/tpbi.2000.1508

[34] Pritchard JK , Przeworski M. Linkage disequilibrium in humans: models and data. Am J Hum Genet 2001;69:1–14. 11410837 10.1086/321275 PMC1226024

[35] Kruglyak L. Prospects for whole-genome linkage disequilibrium mapping of common disease genes. Nat Genet 1999;22:139–144. 10369254 10.1038/9642

[36] McCarthy S , Das S, Kretzschmar W, Delaneau O, Wood AR, Teumer A, Kang HM, Fuchsberger C, Danecek P, Sharp K, Luo Y, Sidore C, Kwong A, Timpson N, Koskinen S, Vrieze S, Scott LJ, Zhang H, Mahajan A, Veldink J, Peters U, Pato C, Duijn CM, Van Gillies CE, Gandin I, Mezzavilla M, Gilly A, Cocca M, Traglia M, Angius A, Barrett JC, Boomsma D, Branham K, Breen G, Brummett CM, Busonero F, Campbell H, Chan A, Chen S, Chew E, Collins FS, Corbin LJ, Smith GD, Dedoussis G, Dorr M, Farmaki AE, Ferrucci L, Forer L, Fraser RM, Gabriel S, Levy S, Groop L, Harrison T, Hattersley A, Holmen OL, Hveem K, Kretzler M, Lee JC, McGue M, Meitinger T, Melzer D, Min JL, Mohlke KL, Vincent JB, Nauck M, Nickerson D, Palotie A, Pato M, Pirastu N, McInnis M, Richards JB, Sala C, Salomaa V, Schlessinger D, Schoenherr S, Slagboom PE, Small K, Spector T, Stambolian D, Tuke M, Tuomilehto J, Berg Lh VD, Rheenen WV, Volker U, Wijmenga C, Toniolo D, Zeggini E, Gasparini P, Sampson MG, Wilson JF, Frayling T, Bakker PD, Swertz MA, McCarroll S, Kooperberg C, Dekker A, Altshuler D, Willer C, Iacono W, Ripatti S, Soranzo N, Walter K, Swaroop A, Cucca F, Anderson CA, Myers RM, Boehnke M, McCarthy MI, Durbin R, Abecasis G, Marchini J; Haplotype Reference Consortium. A reference panel of 64,976 haplotypes for genotype imputation. Nat Genet 2016;48:1279–1283. 27548312 10.1038/ng.3643 PMC5388176

[37] Kamstrup PR , Tybjærg-Hansen A, Steffensen R, Nordestgaard BG. Genetically elevated lipoprotein (a). J Am Med Assoc 2009;301:2331–2339. 10.1001/jama.2009.801 19509380

[38] Hazarika S , Annex BH. Biomarkers and genetics in peripheral artery disease. Clin Chem 2017;63:236–244. 27872083 10.1373/clinchem.2016.263798 PMC5475367

[39] Mack S , Coassin S, Rueedi R, Yousri NA, Seppälä I, Gieger C, Schönherr S, Forer L, Erhart G, Marques-Vidal P, Ried JS, Waeber G, Bergmann S, Dähnhardt D, Stöckl A, Raitakari OT, Kähönen M, Peters A, Meitinger T, Strauch K, Kedenko L, Paulweber B, Lehtimäki T, Hunt SC, Vollenweider P, Lamina C, Kronenberg F; KORA-Study Group. A genome-wide association meta-analysis on lipoprotein (a) concentrations adjusted for apolipoprotein (a) isoforms. J Lipid Res 2017;58:1834–1844. 28512139 10.1194/jlr.M076232 PMC5580897

[40] Guan W , Cao J, Steffen BT, Post WS, Stein JH, Tattersall MC, Kaufman JD, McConnell JP, Hoefner DM, Warnick R, Tsai MY. Race is a key variable in assigning lipoprotein(a) cutoff values for coronary heart disease risk assessment: the multi-ethnic study of atherosclerosis. Arterioscler Thromb Vasc Biol 2015;35:996–1001. 25810300 10.1161/ATVBAHA.114.304785 PMC4377643

[41] Schmidt K , Noureen A, Kronenberg F, Utermann G. Structure, function, and genetics of lipoprotein (a). J Lipid Res 2016;57:1339–1359. 27074913 10.1194/jlr.R067314 PMC4959873

[42] Kronenberg F. Human genetics and the causal role of lipoprotein(a) for various diseases. Cardiovasc Drugs Ther 2016;30:87–100. 26896185 10.1007/s10557-016-6648-3 PMC4789197

[43] Brunner C , Lobentanz EM, Pethö-Schramm A, Ernst A, Kang C, Dieplinger H, Müller HJ, Utermann G. The number of identical kringle IV repeats in apolipoprotein(a) affects its processing and secretion by HepG2 cells. J Biol Chem 1996;271:32403–32410. 8943305 10.1074/jbc.271.50.32403

[44] Zekavat SM , Ruotsalainen S, Handsaker RE, Alver M, Bloom J, Poterba T, Seed C, Ernst J, Chaffin M, Engreitz J, Peloso GM, Manichaikul A, Yang C, Ryan KA, Fu M, Johnson WC, Tsai M, Budoff M, Ramachandran VS, Cupples LA, Rotter JI, Rich SS, Post W, Mitchell BD, Correa A, Metspalu A, Wilson JG, Salomaa V, Kellis M, Daly MJ, Neale BM, McCarroll S, Surakka I, Esko T, Ganna A, Ripatti S, Kathiresan S, Natarajan P, Abe N, Abecasis G, Albert C, Allred NP, Almasy L, Alonso A, Ament S, Anderson P, Anugu P, Applebaum-Bowden D, Arking D, Arnett DK, Ashley-Koch A, Aslibekyan S, Assimes T, Auer P, Avramopoulos D, Barnard J, Barnes K, Barr RG, Barron-Casella E, Beaty T, Becker D, Becker L, Beer R, Begum F, Beitelshees A, Benjamin E, Bezerra M, Bielak L, Bis J, Blackwell T, Blangero J, Boerwinkle E, Borecki I, Bowler R, Brody J, Broeckel U, Broome J, Bunting K, Burchard E, Cardwell J, Carty C, Casaburi R, Casella J, Chang C, Chasman D, Chavan S, Chen BJ, Chen WM, Chen YDI, Cho M, Choi SH, Chuang LM, Chung M, Cornell E, Crandall C, Crapo J, Curran J, Curtis J, Custer B, Damcott C, Darbar D, Das S, David S, Davis C, Daya M, Andrade M, De Debaun M, Deka R, Demeo D, Devine S, Do R, Duan Q, Duggirala R, Durda P, Dutcher S, Eaton C, Ekunwe L, Ellinor P, Emery L, Farber C, Farnam L, Fingerlin T, Flickinger M, Fornage M, Franceschini N, Fullerton SM, Fulton L, Gabriel S, Gan W, Gao Y, Gass M, Gelb B, Geng X, Germer S, Gignoux C, Gladwin M, Glahn D, Gogarten S, Gong DW, Goring H, Gu CC, Guan Y, Guo X, Haessler J, Hall M, Harris D, Hawley N, He J, Heavner B, Heckbert S, Hernandez R, Herrington D, Hersh C, Hidalgo B, Hixson J, Hokanson J, Hong E, Hoth K, Hsiung C, Huston H, Hwu CM, Irvin MR, Jackson R, Jain D, Jaquish C, Jhun MA, Johnsen J, Johnson A, Johnston R, Jones K, Kang HM, Kaplan R, Kardia S, Kaufman L, Kelly S, Kenny E, Kessler M, Khan A, Kinney G, Konkle B, Kooperberg C, Kramer H, Krauter S, Lange C, Lange E, Lange L, Laurie C, Laurie C, Leboff M, Lee SS, Lee WJ, Lefaive J, Levine D, Levy D, Lewis J, Li Y, Lin H, Lin KH, Liu S, Liu Y, Loos R, Lubitz S, Lunetta K, Luo J, Mahaney M, Make B, Manson JA, Margolin L, Martin L, Mathai S, Mathias R, McArdle P, McDonald ML, McFarland S, McGarvey S, Mei H, Meyers DA, Mikulla J, Min N, Minear M, Minster RL, Montasser ME, Musani S, Mwasongwe S, Mychaleckyj JC, Nadkarni G, Naik R, Nekhai S, Nickerson D, North K, O’connell J, O’connor T, Ochs-Balcom H, Pankow J, Papanicolaou G, Parker M, Parsa A, Penchev S, Peralta JM, Perez M, Perry J, Peters U, Peyser P, Phillips L, Phillips S, Pollin T, Becker JP, Boorgula MP, Preuss M, Prokopenko D, Psaty B, Qasba P, Qiao D, Qin Z, Rafaels N, Raffield L, Rao DC, Rasmussen-Torvik L, Ratan A, Redline S, Reed R, Regan E, Reiner A, Rice K, Roden D, Roselli C, Ruczinski I, Russell P, Ruuska S, Sakornsakolpat P, Salimi S, Salzberg S, Sandow K, Sankaran V, Scheller C, Schmidt E, Schwander K, Schwartz D, Sciurba F, Seidman C, Sheehan V, Shetty A, Shetty A, Sheu WHH, Shoemaker MB, Silver B, Silverman E, Smith J, Smith J, Smith N, Smith T, Smoller S, Snively B, Sofer T, Sotoodehnia N, Stilp A, Streeten E, Sung YJ, Sylvia J, Szpiro A, Sztalryd C, Taliun D, Tang H, Taub M, Taylor K, Taylor S, Telen M, Thornton TA, Tinker L, Tirschwell D, Tiwari H, Tracy R, Vaidya D, Vandehaar P, Vrieze S, Walker T, Wallace R, Walts A, Wang WE, Watson FF, Weeks K, Weir DE, Weiss B, Weng S, Willer LC, Williams C, Williams K, Wilson LK, Wong C, Xu Q, Yanek H, Yang L, Yang I, Zaghloul R, Zhang N, Zhao Y, Zhao SX, Zheng W, Zhi X, Zhou D, Zody X, Zoellner M, S. Deep coverage whole genome sequences and plasma lipoprotein(a) in individuals of European and African ancestries. Nat Commun 2018;9:1–14. 29973585 10.1038/s41467-018-04668-w PMC6031652

[45] Kichaev G , Bhatia G, Loh PR, Gazal S, Burch K, Freund MK, Schoech A, Pasaniuc B, Price AL. Leveraging polygenic functional enrichment to improve GWAS power. Am J Hum Genet 2019;104:65–75. 30595370 10.1016/j.ajhg.2018.11.008 PMC6323418

[46] Fish AE , Capra JA, Bush WS. Are interactions between cis-regulatory variants evidence for biological epistasis or statistical artifacts? Am J Hum Genet 2016;99:817–830. 27640306 10.1016/j.ajhg.2016.07.022 PMC5065654

[47] Gusareva ES , Steen KV. Practical aspects of genome-wide association interaction analysis. Hum Genet 2014;133:1343–1358. 25164382 10.1007/s00439-014-1480-y

[48] Sun X , Lu Q, Mukheerjee S, Crane PK, Elston R, Ritchie MD. Analysis pipeline for the epistasis search - statistical versus biological filtering. Front Genet 2014;5:1–7. 24817878 10.3389/fgene.2014.00106 PMC4012196

[49] Kronenberg F. Prediction of cardiovascular risk by Lp(a) concentrations or genetic variants within the LPA gene region. Clin Res Cardiol Suppl 2019;14:5–12. 30859385 10.1007/s11789-019-00093-5

[50] Lambert SA , Abraham G, Inouye M. Towards clinical utility of polygenic risk scores. Hum Mol Genet 2019;28:R133–R142. 31363735 10.1093/hmg/ddz187

[51] Moore JH , Williams SM. Epistasis and its implications for personal genetics. Am J Hum Genet 2009;85:309–320. 19733727 10.1016/j.ajhg.2009.08.006 PMC2771593

[52] Tsimikas S , Karwatowska-Prokopczuk E, Gouni-Berthold I, Tardif JC, Baum SJ, Steinhagen-Thiessen E, Shapiro MD, Stroes ES, Moriarty PM, Nordestgaard BG, Xia S, Guerriero J, Viney NJ, O’Dea L, Witztum JL. Lipoprotein(a) reduction in persons with cardiovascular disease. N Engl J Med 2020;382:244–255. 31893580 10.1056/NEJMoa1905239

[53] Bessonov K , Gusareva ES, Steen KV. A cautionary note on the impact of protocol changes for genome-wide association SNP × SNP interaction studies: an example on ankylosing spondylitis. Hum Genet 2015;134:761–773. 25939665 10.1007/s00439-015-1560-7

