# Supplementary material for: Cis-epistasis at the LPA locus and risk of cardiovascular diseases
Source: Cardiovasc Res. 2021 Apr 20;118(4):1088–102. doi: 10.1093/cvr/cvab136 (PMC8930071; doi:10.1093/cvr/cvab136)
Supplement: cvab136_Supplementary_Data [file cvab136_supplementary_data.zip › supplementary_methods_cardiovascular_research_ref.pdf]

Cis-epistasis at the *LPA* locus and risk of cardiovascular diseases

Lingyao Zeng+, Sylvain Moser+, Nazanin Mirza-Schreiber+, Claudia Lamina, Stefan Coassin, Christopher P. Nelson, Tarmo Annilo, Oscar Franzén, Marcus E. Kleber, Salome Mack, Till F. M. Andlauer, Beibei Jiang, Barbara Stiller, Ling Li, Christina Willenborg, Matthias Munz, Thorsten Kessler, Adnan Kastrati, Karl-Ludwig Laugwitz, Jeanette Erdmann, Susanne Moebus, Markus M. Nöthen, Annette Peters, Konstantin Strauch, Martina Müller-Nurasyid, Christian Gieger, Thomas Meitinger, Elisabeth Steinhagen-Thiessen, Winfried März, Andres Metspalu, Johan L. M. Björkegren, Nilesh J. Samani, Florian Kronenberg, Bertram Müller-Myhsok* & Heribert Schunkert*

SUPPLEMENTAL MATERIAL

Table of Contents

Supplementary Methods 2

KORA F3/F4 studies:....................2

STARNET-Study 2

Genotype processing for 10 CAD case-control studies....................3

Sample QC for the UKBB replication dataset:....................4

Definition of window size for the broad-sense CAD susceptibility regions 5

Likelihood ratio test based forward model selection procedure 5

Relative effect sizes and analyses of intermediate traits 6

Relative effect size for three-SNP haplotypes....................6

Haplotypes vs Genotypes model comparison:....................7

Supplementary Figures: 8

Supplementary Figure 1 cis SNP-pair prioritization of statistical interactions of CAD....................8

Supplementary Figure 2: Increased variance explained by physical expansion around the known CAD lead SNPs reported from GWAS studies. 9

References 9

Supplementary Tables 12-29

Supplementary Methods

KORA F3/F4 studies:

Individual-level genotypes were obtained from population studies from Augsburg, Germany[1]: KORA F3 and KORA F4[2,3]. The KORA F3 study, conducted in the years 2004/05, is a population-based sample from the general population living in the region of Augsburg, Southern Germany, which has evolved from the WHO MONICA study (Monitoring of Trends and Determinants of Cardiovascular Disease). The KORA F4 survey is an independent, non-overlapping sample drawn from the same population in the years 2006/08. The lipid measurements included total lipoprotein(a) [Lp(a)] levels and the number of Kringle repeats of the Lp(a) protein, determined by Western blotting[4]. Apo(a) isoforms were determined by sodium dodecyl sulfate-agarose gel electrophoresis (SDS agarose) under reducing conditions as described in Kronenberg et al[55] .Electrophoresis was followed by immunoblotting using the monoclonal antibody 1A2 for detection of apo(a) isoforms. Given the large number of alleles present in the population, >90% of the individuals in a population are heterozygous on DNA level. However, only about 60-70% present both isoforms in plasma. Larger isoforms tend to be non-expressed due to overly long residence in the endoplasmatic reticulum. Short isoforms are produced more efficiently per time unit and thus expressed at a higher level, commonly contributing to a higher extent to the circulating Lp(a) and thus present a stronger band in the Western blot (>50% of the total intensities of both band). In this study, all statistical models use the predominantly expressed isoform for isoform-based adjustment. These studies obtained institutional review board approval from their local Ethical Committees and were performed in accordance with the 1964 Helsinki Declaration and its later amendments. All patients gave prior written consent.

STARNET-Study

RNAseq data were generated from liver tissue of 522 CABG CAD patients from the Stockholm-Tartu Reverse Network Engineering Task (STARNET) study[3]. All patients were Caucasian (30% females), 27% had diabetes, 77% hypertension, 68% hyperlipidemia, and 37% a myocardial infarction before the age of 60. Patients diagnosed with CAD who were eligible for open-thorax surgery at the Department of Cardiac Surgery, Tartu University Hospital, were enrolled. A 2–3 cm incision in the diaphragm was made to access the peritoneal cavity. This incision was placed to enable direct access to the outer edge of the left lateral liver lobe, from

which a 3–5 mm3 biopsy was obtained. The liver incision was sutured to control any bleeding. Samples were sequenced on Illumina HiSeq with a single-end read length of 50 or 100 base pairs. DNA and RNA qualities were assessed with the Agilent 2100 Bioanalyzer system (Agilent Technologies, Palo Alto, CA). Detailed procedures for genotype and RNA processing, including QC and imputation steps, are provided in Franzén et al. 2016[3]. Informed consent was obtained from all subjects (Ethics Approvals Dnr 154/7 and 188/M-12). This study obtained institutional review board approval from its local Ethical Committees and was performed in accordance with the 1964 Helsinki Declaration and its later amendments.

Genotype processing for 10 CAD case-control studies

Study	Array Platform
**GerMIFSI**	Affymetrix Mapping 500K Array Set
**GerMIFSII**	Affymetrix Genome-Wide Human SNP Array 6.0
**GerMIFSIII**	Affymetrix Genome-Wide Human SNP Array 5.0/6.0
**GerMIFSIV**	Affymetrix Genome-Wide Human SNP Array 6.0
**GerMIFSV**	Illumina HumanOmniExpress/Omniuni_2.5
**LURIC**	Affymetrix Genome-Wide Human SNP Array 6.0
**WTCCC**	Affymetrix Genome-Wide Human SNP Array 6.0
**MIGEN**	Affymetrix Mapping 500K Array Set
**Cardiogenics**	Illumina Human660W-Quad
**GerMIFSVI**	Illumina PsychChip_v1-1

Supplementary Methods Table 1. Gentoype array platforms of ten CAD case-control studies used in the discovery stage.

The 10 case-control studies of coronary disease were originally genotyped with the corresponding arrays (Supplementary Methods Table 1). The following pre-imputation quality control (QC) criteria were taken: individual call rate >= 0.98, SNP call rate > 0.98, minor allele frequency (MAF) > 0.01, concordant recorded and genotype-derived gender, population outliers excluded (deviate beyond mean ± 5×(standard deviation (SD)) for top two dimensions from the multidimensional scaling (MDS) analysis, PI_HAT < 0.0625 (individuals more distant

away than fourth-degree relatives) in the identity-by-descent (IBD) analysis, heterozygosity rate within mean ± 3×SD, and deviation from Hardy-Weinberg Equilibrium (HWE) p > 1e-6.

After genotype QC, we used all individuals from the 1000 Genomes Phase 1 Version 3 reference panel (1000G) to impute the genotypes. Haplotypes were firstly pre-phased from genotypes with SHAPEIT2 haplotype estimation tool to generate the best guess haplotypes based on the given genotypes. Then the best guess haplotypes were forwarded to IMPUTE2 for imputation. Finally, the following post-imputation QC criteria were taken: SNP call rate > 0.98, MAF > 0.05, Hardy-Weinberg p > 1e-5, INFO score >= 0.8.

In the process of our work, the HRC reference dataset[5] was released as the largest available haplotype reference panel for imputation of variants in populations of European ancestry. The coverage of SNPs in our data based on HRC imputation were higher compared to 1000G, thus we repeated the same analysis based the HRC v1.6 imputation, with the hope of fine-mapping the lead- SNP-pair. The same pre-imputation QC criteria were applied as for 1000G. The imputation procedures were conducted through Sanger Imputation Server (https://www.sanger.ac.uk/science/tools/sanger-imputation-service). The following post-imputation QC criteria were applied: SNP call rate > 0.98, MAF > 0.05, Hardy-Weinberg p > 1e-5, INFO score >= 0.8. Moreover, a threshold of PI_HAT < 0.0625 (individuals more distant away than fourth-degree relatives) was applied again on the identity-by-descent (IBD) matrix calculated on the imputed data. This allowed to remove samples which became closer than 4th degree relatives because of the imputation.

Sample QC for the UKBB replication dataset:

We obtained imputed data from 3rd release of UK Biobank. The following per-SNP QC were performed: MAF > 0.00001, imputation info > 0.4, SNP-level calling rate > 0.95, HWE with P > 1e-5. We also applied the following the following per-sample QC: individual-level call rate >= 0.98, sex consistence, kinship coefficient < 0.088 and deviation from HWE with P > 1e-5. Moreover, only individuals with European Ancestry were included in the association analysis on CAD risk and Lp(a) to avoid biases due to inter-population differences in the genetic control of Lp(a) levels. Indeed, inter-population differences have been reported between population regarding Lp(a) levels and genetic variants associated to it[6].

Definition of window size for the broad-sense CAD susceptibility regions

We focused our analysis at loci with previous evidence of genome-wide association with CAD in order to restrict the number of variants for testing of statistical epistasis with the aim to enhance speed and the chance of positive finding. Firstly, we defined regions of CAD susceptibility in a broad-sense, i.e., flanking regions of known CAD risk loci, given that multiple independent signals at the surrounding region of known CAD lead-SNPs could explain additional heritability to CAD[7,8]. To achieve this, we collected the lead SNPs from the 56 published CAD susceptibility loci[7,9], and calculated the SNP-based heritability for all SNPs within a certain flanking range of known loci, from ±100kb to ±1mb progressively with steps of 100kb.For heritability calculation, all variants in the flanking region around the lead SNPs at 56 loci with available genotypes in nine CAD case-controls studies were extracted (GerMIFSVI not included at this time). Imputed genotypes or proxy variants were used where necessary. The LDAK tool[10] was used to calculate the LD-adjusted kinship matrix among all individuals, which was then forwarded to software GCTA[11] to estimate the SNP-based heritability of CAD in the measurement of the total variance explained in liability model (assuming a prevalence of CAD as 5%). As expected, an incremental increase in the heritability was observed with the enlargement of the flanking region (Supplementary Fig 3). The increase of heritability explained by the regions increased profoundly from ±100kb until ±500kb but largely attenuated afterwards. Therefore, we decided ±500kb as a balanced threshold for the flanking range surrounding the known loci (i.e., the broad-sense CAD susceptibility regions), which could meanwhile maximize the heritability covered by the regions and minimize the computational burden.

Likelihood ratio test based forward model selection procedure

In order to determine if epistasis effect were present at the LPA locus in addition to the marginal effect of rs140570886 , rs1652507 and rs9458001 we performed a forward model selection based on LRT. We started with a base model including only the principal components of the genetic variance. We then added the following term in a stepwise manner and computed a LRT with respect to the previous model:

1) Additive effect of rs140570886
2) Additive effect of rs9458001 and rs1652501
3) Interaction between rs9458001:rs1652507
4) All possible 3-way interactions between rs140570886: rs9458001:rs1652507

5) Additive effect of rs3798220
6) All possible 4-way interactions between rs140570886: rs94580051rs1652507:rs379822

After having determined the 4th model as the best one (i.e the last one to be significantly better than its precedent) we computed a likelihood ratio test between model 4 and 1 to assess the gain in model fit due to the incorporation of these variables. This analysis was carried on the merged dataset of the 10 CAD studies and the UKBB dataset together. Every model also included the principal components of the genetic variance (computed on the merged dataset of the 10 CAD studies together and on the UKBB dataset separately) and a factor indicating the study. The anova() function from base R was used for the computation.

Relative effect sizes and analyses of intermediate traits

In order to calculate genotypic effect sizes for different genotypes, regression analysis was performed in R using the same model as for the minor allele dosage but coding the genotypes according to their posterior probabilities from the imputation, with the major allele homozygous genotype as the reference.

For the association analysis on intermediate traits, regression analyses were performed using R based on the same genetic model as for CAD after replacement of the dependent variable y with the corresponding intermediate traits of interest. Covariates were added where necessary for each intermediate factor. Due to the highly skewed distribution of Lp(a), inverse normal transformation was applied to the Lp(a) concentration. The analysis of Lp(a) levels was performed separately in KORA F3 and F4, followed by meta-analysis.

Relative effect size for three-SNP haplotypes

Given that only genotypes, rather than haplotypes, are directly measured in genotyping arrays, we estimated haplotypes via linkage phasing of the SNP genotypes for ambiguous (heterozygous at both SNPs) haplotypes using the R package *hapassoc*. The haplotypes association analysis was then performed using the *happassoc* function fitting a generalized linear model (GLM) [Eq (5)] using the expectation-maximization (EM) algorithm.

$$y \sim b_0 + b_{h1} \times haplo1 + b_{h2} \times haplo2 + b_{h3} \times haplo3 + ... + b_{h8} \times haplo8 + b_{c1} \times \mathrm{cov}_1 + b_{c2} \times \mathrm{cov}_2 + ... + b_{c10} \times \mathrm{cov}_{10} \quad (5)$$

The most frequent haplotype was set as the reference and relative effect sizes were computed for the seven other haplotypes. The ten first MDS components of the genetic relationship matrix, computed on the merged dataset of the ten CAD studies, were used as covariates in the

GLM. We tried to replicate this analysis in the UKBB dataset, however, the EM algorithm failed to converge on this very big dataset.

Haplotypes vs Genotypes model comparison:

In order to directly test whether a putative haplotype tagged by the combination of 140570886 , rs1652507 and rs9458001 provides a better fit than a model including genotypes and interactions, we compared the following models based on Akaike Information Criterion (AIC) , Bayseian Information Criterion (BIC) and LRT: (* represents is a shortcut representation for main effect and interractions)

Model genotype: CAD ~ rs9458001 + rs1652507 + rs140570886 + covariates

Model haplotypes: CAD ~ hap1 + hap2+hap3+hap4+hap5+hap6+hap7+hap8 + covariates

Model genotype + interaction: CAD ~rs9458001 * rs1652507 * rs140570886 + covariates

Model haplotype + interaction: CAD ~ hap1 + hap2+hap3+hap4+hap5+hap6+hap7+hap8 + rs9458001 * rs1652507 * rs140570886 + covariates

(* is a shortcut representation for main effect and interractions)

The genotype variable in these models were coded as minor allele dosage, similar to the other analysis in this study. Haplotypes were calculated using the R package *haplo.stats*. Pseudo-individuals were created for individuals whose haplotypes couldn't be unambiguously estimated. The posterior probabilities of each pseudo-individual's haplotype were then used as weights in weighted logistic regression model in R. LRT was moreover performed for each pair of nested models. This analysis was carried on the merged dataset of the 10 CAD studies and the UKBB dataset together. Every model also included the principal components of the genetic variance (computed on the merged dataset of the 10 CAD studies together and on the UKBB dataset separately) and a factor indicating the study.

Supplementary Figures:

a

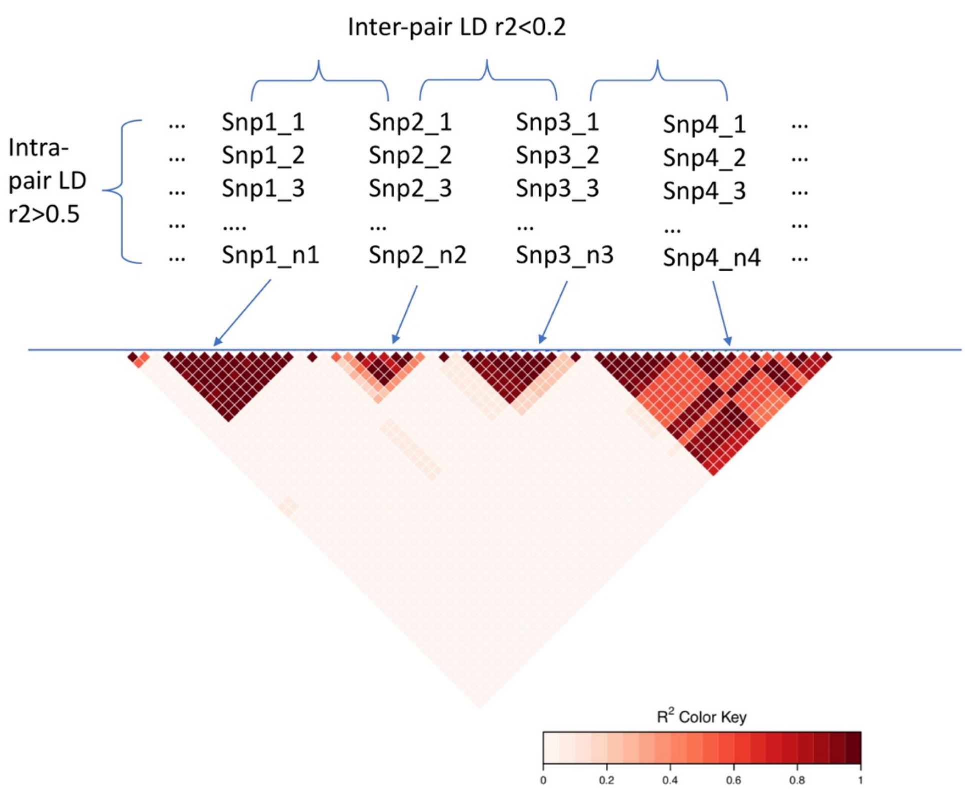

Supplementary Figure 1

cis SNP-pair prioritization of statistical interactions of CAD.

LD heatmap showing distinct LD blocks along the genome in the vicinity region. Panel **a.** characterizes the methods of defining statistical cis-epistasis. Statistical epistasis pairs were chosen between two non-LD singleton lead SNPs. LD-independent blocks were identified for all variants in the vicinity region. Those with a LD of $r^2 > 0.5$ were grouped into the same SNP bunch, while those with $r^2 < 0.2$ were considered as distinct independent LD blocks. LD singleton (with weak association r^2 between 0.2 - 0.5 with any other) were not of our interest for the consideration of replication and interpretation reason.

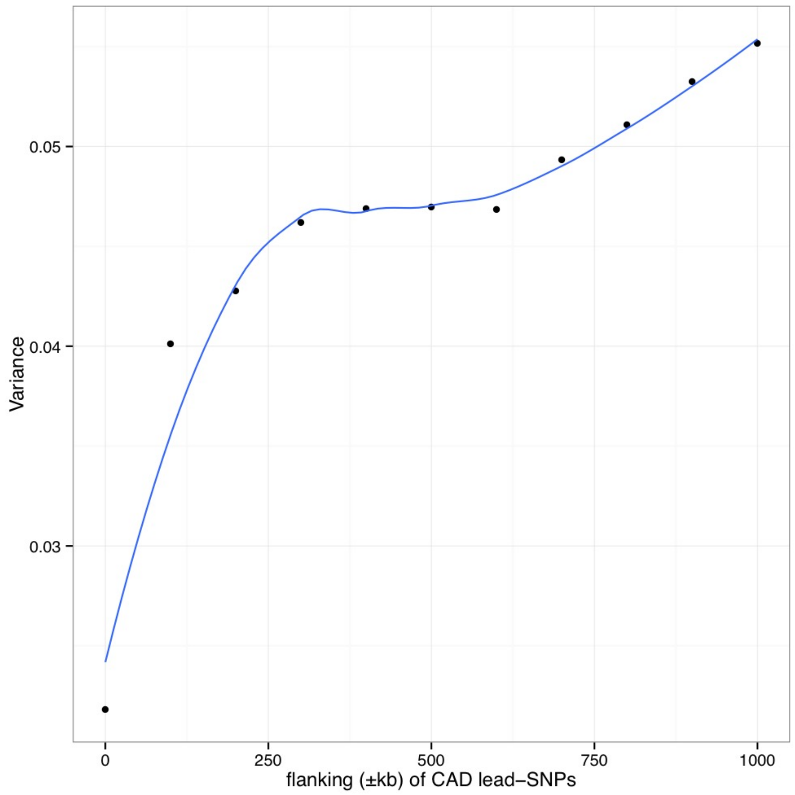

Supplementary Figure 2: Increased variance explained by physical expansion around the known CAD lead SNPs reported from GWAS studies.

All variants in the flanking region around the lead SNPs at 56 loci with available genotypes in nine CAD case-controls studies were extracted. LDAK tool was used to calculate the LD-adjusted kinship matrix among all individuals, which was then forwarded to GCTA to estimate the SNP- based heritability of CAD in the measurement of the total variance explained in liability model (assuming a prevalence of CAD as 5%). The x-axis represents the flanking range of known loci, from ±100kb to ±1mb progressively with steps of 100kb. The y-axis represents the variance of CAD risk that could be explained by the given SNPs in the liability model. Indeed, variance explained including the flanking regions achieved 0.47 around the step at ±500kb, while variance explained of the lead SNPs only (±0kb) was 0.22, which was only a 46.5% proportion (0.22/0.47).

References

1. Heid IM, Boes E, Müller M, Kollerits B, Lamina C, Coassin S, Gieger C, Döring A, Klopp N, Frikke-Schmidt R, Tybjaerg-Hansen A, Brandstätter A, Luchner A, Meitinger T, Wichmann HE, Kronenberg F. Genome-wide association analysis of high-density

lipoprotein cholesterol in the population-based KORA study sheds new light on intergenic regions. *Circ Cardiovasc Genet* 2008;**1**:10–20.

2. Holle R, Happich M, Löwel H, Wichmann HE. KORA - A research platform for population based health research. *Gesundheitswesen* 2005;**67**:S19-25.

3. Franzén O, Ermel R, Cohain A, Akers NK, Narzo A Di, Talukdar HA, Foroughi-Asl H, Giambartolomei C, Fullard JF, Sukhavasi K, Köks S, Gan LM, Giannarelli C, Kovacic JC, Betsholtz C, Losic B, Michoel T, Hao K, Roussos P, Skogsberg J, Ruusalepp A, Schadt EE, Björkegren JLM. Cardiometabolic risk loci share downstream cis- and trans-gene regulation across tissues and diseases. *Science (80-)* 2016;**353**:827–830.

4. Mack S, Coassin S, Rueedi R, Yousri NA, Seppälä I, Gieger C, Schönherr S, Forer L, Erhart G, Marques-Vidal P, Ried JS, Waeber G, Bergmann S, Dähnhardt D, Stöckl A, Raitakari OT, Kähönen M, Peters A, Meitinger T, Strauch K, Kedenko L, Paulweber B, Lehtimäki T, Hunt SC, Vollenweider P, Lamina C, Kronenberg F. A genome-wide association meta-analysis on lipoprotein (a) concentrations adjusted for apolipoprotein (a) isoforms. *J Lipid Res* 2017;**58**:1834–1844.

5. McCarthy S, Das S, Kretzschmar W, Delaneau O, Wood AR, Teumer A, Kang HM, Fuchsberger C, Danecek P, Sharp K, Luo Y, Sidore C, Kwong A, Timpson N, Koskinen S, Vrieze S, Scott LJ, Zhang H, Mahajan A, Veldink J, Peters U, Pato C, Duijn CM Van, Gillies CE, Gandin I, Mezzavilla M, Gilly A, Cocca M, Traglia M, Angius A, Barrett JC, Boomsma D, Branham K, Breen G, Brummett CM, Busonero F, Campbell H, Chan A, Chen S, Chew E, Collins FS, Corbin LJ, Smith GD, Dedoussis G, Dorr M, Farmaki AE, Ferrucci L, Forer L, Fraser RM, Gabriel S, Levy S, Groop L, Harrison T, Hattersley A, Holmen OL, Hveem K, Kretzler M, Lee JC, McGue M, Meitinger T, Melzer D, Min JL, Mohlke KL, Vincent JB, Nauck M, Nickerson D, Palotie A, Pato M, Pirastu N, McInnis M, Richards JB, Sala C, Salomaa V, Schlessinger D, Schoenherr S, Slagboom PE, Small K, Spector T, Stambolian D, Tuke M, Tuomilehto J, Berg LH Van Den, Rheenen W Van, Volker U, Wijmenga C, Toniolo D, Zeggini E, Gasparini P, Sampson MG, Wilson JF, Frayling T, Bakker PIW De, Swertz MA, McCarroll S, Kooperberg C, Dekker A, Altshuler D, Willer C, Iacono W, Ripatti S, Soranzo N, Walter K, Swaroop A, Cucca F, Anderson CA, Myers RM, Boehnke M, McCarthy MI, Durbin R, Abecasis G, Marchini J. A reference panel of 64,976 haplotypes for genotype imputation. *Nat Genet* 2016;**48**:1279–1283.

6. Dumitrescu L, Glenn K, Brown-Gentry K, Shephard C, Wong M, Rieder MJ, Smith JD, Nickerson DA, Crawford DC. Variation in LPA is associated with Lp(a) levels in three

populations from the third National Health and Nutrition Examination Survey. *PLoS One* 2011;**6**.

7. Nikpay M, Goel A, Won HH, Hall LM, Willenborg C, Kanoni S, Saleheen D, Kyriakou T, Nelson CP, CHopewell J, Webb TR, Zeng L, Dehghan A, Alver M, MArmasu S, Auro K, Bjonnes A, Chasman DI, Chen S, Ford I, Franceschini N, Gieger C, Grace C, Gustafsson S, Huang J, Hwang SJ, Kim YK, Kleber ME, Lau KW, Lu X, Lu Y, Lyytikäinen LP, Mihailov E, Morrison AC, Pervjakova N, Qu L, Rose LM, Salfati E, Saxena R, Scholz M, Smith A V., Tikkanen E, Uitterlinden A, Yang X, Zhang W, Zhao W, Andrade M De, Vries PS De, Zuydam NR Van, Anand SS, Bertram L, Beutner F, Dedoussis G, Frossard P, Gauguier D, Goodall AH, Gottesman O, Haber M, Han BG, Huang J, Jalilzadeh S, Kessler T, König IR, Lannfelt L, Lieb W, Lind L, MLindgren C, Lokki ML, Magnusson PK, Mallick NH, Mehra N, Meitinger T, Memon FUR, Morris AP, Nieminen MS, Pedersen NL, Peters A, Rallidis LS, Rasheed A, Samuel M, Shah SH, Sinisalo J, EStirrups K, Trompet S, Wang L, Zaman KS, Ardissino D, Boerwinkle E, Borecki IB, Bottinger EP, Buring JE, Chambers JC, Collins R, Cupples L, Danesh J, Demuth I, Elosua R, Epstein SE, Esko T, Feitosa MF, Franco OH, Franzosi MG, Granger CB, Gu D, Gudnason V, SHall A, Hamsten A, Harris TB, LHazen S, Hengstenberg C, Hofman A, Ingelsson E, Iribarren C, Jukema JW, Karhunen PJ, Kim BJ, Kooner JS, Kullo IJ, Lehtimäki T, Loos RJF, Melander O, Metspalu A, März W, Palmer CN, Perola M, Quertermous T, Rader DJ, Ridker PM, Ripatti S, Roberts R, Salomaa V, Sanghera DK, Schwartz SM, Seedorf U, Stewart AF, Stott DJ, Thiery J, Zalloua PA, O'Donnell CJ, Reilly MP, Assimes TL, Thompson JR, Erdmann J, Clarke R, Watkins H, Kathiresan S, McPherson R, Deloukas P, Schunkert H, Samani NJ, Farrall M. A comprehensive 1000 Genomes-based genome-wide association meta-analysis of coronary artery disease. *Nat Genet* 2015;**47**:1121–1130.

8. Nelson CP, Goel A, Butterworth AS, Kanoni S, Webb TR, Marouli E, Zeng L, Ntalla I, Lai FY, Hopewell JC, Giannakopoulou O, Jiang T, Hamby SE, Angelantonio E Di, Assimes TL, Bottinger EP, Chambers JC, Clarke R, Palmer CNA, Cubbon RM, Ellinor P, Ermel R, Evangelou E, Franks PW, Grace C, Gu D, Hingorani AD, Howson JMM, Ingelsson E, Kastrati A, Kessler T, Kyriakou T, Lehtimäki T, Lu X, Lu Y, März W, McPherson R, Metspalu A, Pujades-Rodriguez M, Ruusalepp A, Schadt EE, Schmidt AF, Sweeting MJ, Zalloua PA, Alghalayini K, Keavney BD, Kooner JS, Loos RJF, Patel RS, Rutter MK, Tomaszewski M, Tzoulaki I, Zeggini E, Erdmann J, Dedoussis G, Björkegren JLM, Schunkert H, Farrall M, Danesh J, Samani NJ, Watkins H, Deloukas

P. Association analyses based on false discovery rate implicate new loci for coronary artery disease. *Nat Genet* 2017;**49**:1385–1391.

9. Deloukas P, Kanoni S, Willenborg C, Farrall M, Assimes TL, Thompson JR, Ingelsson E, Saleheen D, Erdmann J, Goldstein BA, Stirrups K, König IR, Cazier JB, Johansson Å, Hall AS, Lee JY, Willer CJ, Chambers JC, Esko T, Folkersen L, Goel A, Grundberg E, Havulinna AS, Ho WK, Hopewell JC, Eriksson N, Kleber ME, Kristiansson K, Lundmark P, Lyytikäinen LP, Rafelt S, Shungin D, Strawbridge RJ, Thorleifsson G, Tikkanen E, Zuydam N Van, Voight BF, Waite LL, Zhang W, Ziegler A, Absher D, Altshuler D, Balmforth AJ, Barroso I, Braund PS, Burgdorf C, Claudi-Boehm S, Cox D, Dimitriou M, Do R, Doney ASF, Mokhtari NE El, Eriksson P, Fischer K, Fontanillas P, Franco-Cereceda A, Gigante B, Groop L, Gustafsson S, Hager J, Hallmans G, Han BG, Hunt SE, Kang HM, Illig T, Kessler T, Knowles JW, Kolovou G, Kuusisto J, Langenberg C, Langford C, Leander K, Lokki ML, Lundmark A, McCarthy MI, Meisinger C, Melander O, Mihailov E, Maouche S, Morris AD, Müller-Nurasyid M, Nikus K, Peden JF, Rayner NW, Rasheed A, Rosinger S, Rubin D, Rumpf MP, Schäfer A, Sivananthan M, Song C, Stewart AFR, Tan ST, Thorgeirsson G, Schoot CE Van Der, Wagner PJ, Wells GA, Wild PS, Yang TP, Amouyel P, Arveiler D, Basart H, Boehnke M, Boerwinkle E, Brambilla P, Cambien F, Cupples AL, Faire U De, Dehghan A, Diemert P, Epstein SE, Evans A, Ferrario MM, Ferrières J, Gauguier D, Go AS, Goodall AH, Gudnason V, Hazen SL, Holm H, Iribarren C, Jang Y, Kähönen M, Kee F, Kim HS, Klopp N, Koenig W, Kratzer W, Kuulasmaa K, Laakso M, Laaksonen R, Lee JY, Lind L, Ouwehand WH, Parish S, Park JE, Pedersen NL, Peters A, Quertermous T, Rader DJ, Salomaa V, Schadt E, Shah SH, Sinisalo J, Stark K, Stefansson K, Trégouët DA, Virtamo J, Wallentin L, Wareham N, Zimmermann ME, Nieminen MS, Hengstenberg C, Sandhu MS, Pastinen T, Syvänen AC, Hovingh GK, Dedoussis G, Franks PW, Lehtimäki T, Metspalu A, Zalloua PA, Siegbahn A, Schreiber S, Ripatti S, Blankenberg SS, Perola M, Clarke R, Boehm BO, O'Donnell C, Reilly MP, März W, Collins R, Kathiresan S, Hamsten A, Kooner JS, Thorsteinsdottir U, Danesh J, Palmer CNA, Roberts R, Watkins H, Schunkert H, Samani NJ. Large-scale association analysis identifies new risk loci for coronary artery disease. *Nat Genet* 2013;**45**:25–33.
10. Speed D, Hemani G, Johnson MR, Balding DJ. Improved heritability estimation from genome-wide SNPs. *Am J Hum Genet* 2012;**91**:1011–1021.
11. Yang J, Lee SH, Goddard ME, Visscher PM. GCTA: A tool for genome-wide complex trait analysis. *Am J Hum Genet* 2011;**88**:76–82.

Supplementary Table 1.
Sample size description for all studies providing individual-level genotypes

	Study name	N individuals				
		CAD cases	female(%)	controls	female(%)	all
CAD	*Discovery*					
	**Cardiogenics**	366	0.13114754	401	0.59102244	767
	**GerMIFSI**	621	0.33172303	1518	0.51251647	2139
	**GerMIFSII**	1187	0.20724516	1234	0.47893031	2421
	**GerMIFSIII**	1046	0.20076482	1416	0.48587571	2462
	**GerMIFSIV**	940	0.35212766	1128	0.61258865	2068
	**GerMIFSV**	2389	0.24361658	1535	0.52442997	3924
	**GerMIFSVI**	1634	0.3004896	1177	0.51401869	2811
	**LURIC**	2083	0.25108017	590	0.43220339	2673
	**MIGEN**	2827	0.22285108	2909	0.24097628	5736
	**WTCCC**	1884	0.20859873	2870	0.50836237	4754
	Replication					
	**UK Biobank**	26792	0.31434757	285520	0.55253572	312312
intermediate factors	KORA F3/F4					5953

Supplementary Table 2.
Four candidate SNP pairs showing statistical interactions on CAD risk in 10 studies

	rsID_effectAllele	genomic position	nearest gene	MAF	Known GWAS effect (Nelson et al, 2017) in additive model						
		(chr:bp_hg19)	(location)		OR	OR [95% CI]		p-value			
SNP1	rs1800769_T	chr6:161085267	LPA (UTR5)	0.13	0.99	0.97	1.01	0.3026			
SNP2	rs9458001_A	chr6:161115999	LPA,PLG (intergenic)	0.25	1.01	0.99	1.03	0.2435			
distance SNP1 & SNP2		cis (31kb)									
MAF SNP1 x MAF SNP2				0.0325							
LD SNP1 & SNP2		CAD cases	controls	reference							
	r2	0.00147289	0.0164417	0.0172649							
	D'	0.173989	0.585295	0.594903							
effect sizes for SNP1-SNP2 interaction		SNP1:SNP2									
	data source	model	OR	OR [95% CI]		p-value					
	v1000G	dosage : dosage	1.42	1.29	1.55	**1.75E-13**					
effect sizes of each single SNP for comparison		SNP1					SNP2				
	data source	model	OR	OR [95% CI]		p-value	model	OR	OR [95% CI]		p-value
	v1000G	dosage	0.99	0.94	1.04	0.5915	dosage	1.04	1.00	1.08	0.0823
effect sizes for genotype subgroups (SNP1_SNP2)			subgroup vs reference								
			OR	OR [95% CI]		p-value					
	majority group taken as the reference	CC_GG	1.00	1.00	1.00	/					
	most risky group	TT-GA (actually TT-AA)*	1.70	1.20	2.39	0.0025					
	most protective group	TT_GG	0.82	0.68	0.99	0.0378					

	rsID_effectAllele	genomic position	nearest gene	MAF	Known GWAS effect (Nelson et al, 2017) in additive model						
		(chr:bp_hg19)	(location)		OR	OR [95% CI]		p-value			
SNP1	rs116632378_T	chr6:32293532	C6orf10 (intronic)	0.308	1.00	0.98	1.02	**0.8465**			
SNP2	rs3823438_G	chr6:34962060	ANKS1A (intronic)	0.425	0.99	0.97	1.00	**0.1306**			
distance SNP1 & SNP2		cis (2.7MB)									
MAF SNP1 x MAF SNP2				0.1309							
LD SNP1 & SNP2		CAD cases	controls	reference							
	r2	0.00108229	0.00104019	0.001897							
	D'	0.059381	0.0424859	0.0533678							
effect sizes for SNP1-SNP2 interaction		SNP1:SNP2									
	data source	model	OR	OR [95% CI]		p-value					
	v1000G	dosage : dominant	0.78	0.72	0.85	**3.43E-09**					
effect sizes of each single SNP for comparison		SNP1					SNP2				
	data source	model	OR	OR [95% CI]		p-value	model	OR	OR [95% CI]		p-value
	v1000G	dosage	1.01	0.97	1.05	0.7713	dominant	0.94	0.89	0.99	0.0240
effect sizes for genotype subgroups (SNP1_SNP2)			subgroup vs reference								
			OR	OR [95% CI]		p-value					
	majority group taken as the reference	CC_AA	1.00	1.00	1.00	/					
	most risky group	TT_AA	1.36	1.16	1.60	0.0001					
	most protective group	TT_GG	0.83	0.67	1.03	0.0904					

	rsID_effectAllele	genomic position	nearest gene	MAF	Known GWAS effect (Nelson et al, 2017) in additive model						
		(chr:bp_hg19)	(location)		OR	OR [95% CI]		p-value			
**SNP1**	rs4709404_T	chr6:160591846	22A1,SLC22A2 (interge	0.077	1.05	1.03	1.08	**0.0001**			
**SNP2**	rs1652507_C	chr6:161082461	LPA (intronic)	0.154	0.98	0.96	1.01	**0.1719**			
**distance SNP1 & SNP2**		cis (491kb)									
**MAF SNP1 x MAF SNP2**				0.011858							
		CAD cases	controls	reference							
**LD SNP1 & SNP2**	**r2**	0.0148125	0.000747406	0.0001379							
	**D'**	0.177234	0.0425974	0.102575							
**effect sizes for SNP1-SNP2 interaction**		**SNP1:SNP2**									
	**data source**	model	OR	OR [95% CI]		p-value					
	v1000G	dosage : dosage	1.48	1.30	1.69	5.80E-09					
**effect sizes of each single SNP for comparison**		**SNP1**					**SNP2**				
	**data source**	model	OR	OR [95% CI]		p-value	model	OR	OR [95% CI]		p-value
	v1000G	dosage	1.13	1.05	1.22	0.0007	dosage	0.97	0.92	1.02	0.2115
**effect sizes for genotype subgroups (SNP1_SNP2)**			**subgroup vs reference**								
			OR	OR [95% CI]		p-value					
	majority group taken as the reference	CC_TT	1.00	1.00	1.00	/					
	most risky group	TT_TC (actually TT_CC)*	2.19	1.12	4.29	0.0224					
	most protective group	CC_CC	0.81	0.66	0.99	0.0367					

	rsID_effectAllele	genomic position	nearest gene	MAF	Known GWAS effect (Nelson et al, 2017) in additive model						
		(chr:bp_hg19)	(location)		OR	OR [95% CI]		p-value			
**SNP1**	rs4551571_C	chr1:87614244	C01140 (ncRNA_intro	0.402	1.02	1.00	1.04	**0.0146**			
**SNP2**	rs7451008_C	chr6:20673880	CDKAL1 (intronic)	0.281	1.02	1.00	1.04	**0.0504**			
**distance SNP1 & SNP2**		trans									
**MAF SNP1 x MAF SNP2**				0.112962							
**effect sizes for SNP1-SNP2 interaction**		**SNP1:SNP2**									
	**data source**	model	OR	OR [95% CI]		p-value					
	v1000G	dominant : recessive	0.54	0.44	0.67	**4.64E-09**					
**effect sizes of each single SNP for comparison**		**SNP1**					**SNP2**				
	**data source**	model	OR	OR [95% CI]		p-value	model	OR	OR [95% CI]		p-value
	v1000G	dominant	1.01	0.96	1.06	0.7502	recessive	1.07	0.97	1.18	0.1499
**effect sizes for genotype subgroups (SNP1_SNP2)**			**subgroup vs reference**								
			OR	OR [95% CI]		p-value					
	majority group taken as the reference	GG_TT	1.00	1.00	1.00	/					
	most risky group	GG_CC	1.60	1.35	1.89	2.89E-08					
	most protective group	GC_CC	0.96	0.76	1.22	0.7388					

Notes:
All SNP-pairs identified as candidate statistical epistasis of CAD were summarized in the table. Inclusion criteria (see Methods section for details) :
1) the SNP pair available and the effect of the interaction term showed consistency in at least 8 out of 10 studies, and reached significance level (p<4.61e-9) in either v1000G fixed-effect meta-analysis
2) for cis-pairs, LD r2 < 0.2 between SNP1 and SNP2
3) robust in the conditional analyses conditioned either on a known CAD risk SNP, or SNPs in the flanking ±200kb of the lead SNP.
All alleles are diaplayed as the ones on the positive strand.
Annovar (Wang et al 2010) was utilized to annotate the relative positions and function to the reference gene.
503 European samples from phase3_shapeit2_mvncall_integrated_v5.20130502.genotypes were used to estimate the LD in reference population.
Most risky/protective groups were decided according to the maximum and minumum relative odds ratio (reference groups was set as the carriers with homozygous major alleles for both SNPs)
*altogether 7 rs4709404_TT and rs1652507_CC carriers, who are exclusively CAD cases, thus the odds ratio could not be estimate appropriately.

Supplementary Table 6.

Summary statistics for rs1800769-rs9458001 epistasis on CAD risk using HRC imputation, total Lp(a) level, and LPA gene expression activity

Test item		Odds Ratio (CAD)				
		N individuals / Haplotype frequencies	OR	OR [95% CI]		p-value
**rs1800769 : rs9458001**	**T:A as effect allele**	29755	1.3650	1.2479	1.4932	**1.07E-11**
Single SNP rs1800769 dosage	T as effect allele	29755	0.9789	0.9333	1.0268	0.382
Single SNP rs9458001 dosage	A as effect allele	29755	1.0353	0.9929	1.0796	0.104

Test item		total Lp(a)*			
		N individuals / Haplotype frequencies	beta	se	p-value
**rs1800769 : rs9458001**	**T:A as effect allele**	5953	**0.5806**	**0.0492**	**8.67E-32**
Single SNP rs1800769 dosage	T as effect allele	5953	-0.0693	0.0251	0.005831877
Single SNP rs9458001 dosage	A as effect allele	5953	0.0703	0.0220	0.001377706

Test item		apo(a) isoforms*			
		N individuals / Haplotype frequencies	beta	se	p-value
**rs1800769 : rs9458001**	**T:A as effect allele**	5953	**-3.652127239**	**0.31561887**	**1.22E-30**
Single SNP rs1800769 dosage	T as effect allele	5953	3.140072683	0.16290057	2.31E-80
Single SNP rs9458001 dosage	A as effect allele	5953	-1.957878496	0.14448001	3.17E-41

Test item		total Lp(a)* adjusted for apo(a) isoforms*			
		N individuals / Haplotype frequencies	beta	se	p-value
**rs1800769 : rs9458001**	**T:A as effect allele**	5953	**0.2835**	**0.0424**	**2.59E-11**
Single SNP rs1800769 dosage	T as effect allele	5953	0.1882	0.0219	1.20E-17
Single SNP rs9458001 dosage	A as effect allele	5953	-0.0861	0.0190	5.80E-06

Test item		LPA mRNA			
		N individuals / Haplotype frequencies	beta	se	p-value
**rs1800769 : rs9458001**	**T:A as effect allele**	522	**0.3931**	**0.0682**	**1.41E-08**
Single SNP rs1800769 dosage	T as effect allele	522	0.2456	0.0404	2.28E-09
Single SNP rs9458001 dosage	A as effect allele	522	-0.2629	0.0401	1.30E-10

Note:

rs1652507 was used as proxy for rs1800769 for the CAD analysis

Results on Lp(a) level without and with adjustment of the apo(a) isoforms were calculated based on the KORA cohorts.

Lp(a)*: The original Lp(a) level in mg/dl is highly left-skewed, therefore here raw Lp(a) levels were first inverse normal transformed and then followed with the corresponding analysis.

Supplementary Table 7

Characterization of absolute Lp(a) mg/dl in KORA F3/F4 cohorts

	N individuals	Mean ± SD	25%, 50%, 75% Percentiles
all individuals	4086	22.05 ± 25.38	5.20, 11.53, 29.91
all rs1800769[T] carriers	1192	21.46 ± 27.79	4.96, 10.12, 24.76
all rs9458001[A] carriers	1615	23.00 ± 28.09	5.89, 10.82, 27.49
all rs1800769[C]-rs9458001[A] carriers	1572	22.50 ± 27.49	5.86, 10.72, 26.68
all rs1800769[T]-rs9458001[A] carriers	43	68.30 ± 40.40	46.83, 69.39, 97.97
all rs1800769[C]-rs9458001[G] carriers	3783	21.33 ± 23.55	4.99, 11.81, 29.83
all rs1800769[T]-rs9458001[G] carriers	1149	20.58 ± 26.78	4.86, 9.94, 23.32

Supplementary Table 8.
Replication of rs1800769-rs9458001 epistasis on CAD risk in UK-Biobank

**Cardiovascular trait**	**data source**			**effect sizes for SNP1-SNP2 interaction**					**effect sizes of each single SNP for comparison**									
				**rs1800769[T]:rs9458001[A]**					**rs1800769[T]**					**rs9458001[A]**				
			N cases/controls	model	OR	OR [95% CI]		p-value	model	OR	OR [95% CI]		p-value	model	OR	OR [95% CI]		p-value
	meta-Discovery	**10 studies (v1000G)**	15247/14933		1.42	1.29	1.55	1.75E-13		0.987	0.939	1.036	0.591		1.038	0.995	1.084	0.082
**CAD**		10 studies (vHRC)	14977/14778	dosage : dosage	1.37	1.25	1.49	1.07E-11	dosage	0.979	0.933	1.027	0.38	dosage	1.04	0.993	1.080	1.04E-01
	Replication	**UK-Biobank**	26792/285520		1.15	1.10	1.20	5.67E-10		0.990	0.966	1.014	0.395		1.025	1.004	1.047	0.021
**aortic valve stenosis**	**UK-Biobank**		2023/477496	dosage : dosage	1.47	1.26	1.72	6.95E-07	dosage	0.941	0.863	1.026	0.171	dosage	1.007	0.933	1.087	0.860
**peripheral arterial disease**	**UK-Biobank**		4460/475059	dosage : dosage	1.22	1.10	1.36	2.32E-04	dosage	0.935	0.881	0.991	0.024	dosage	1.034	0.982	1.089	0.203

Note:
rs1652507 was used as proxy for rs1800769 in the 10 studies with HRC imputation and in the independent UKBB , which has LD r2=0.965, D'=0.991, and physical dist=2806 bp
Linkage Disequilibrium was computed on European samples of the 1000 Genome reference panel using the LDmatrix online tool https://ldlink.nci.nih.gov/?tab=ldmatrix

Supplementary Table 9

rs1652507 -rs9458001 interaction and rs140570886 marginal effect on CAD risk and total Lp(a) level specifically in LURIC study

Test item			Odds Ratio (CAD)				Lp(a)*				Odds Ratio (CAD) adjusted by Lp(a)*			
	N CAD cases	N controls	OR	OR [95% CI]		p-value	beta	beta [95%CI]		p-value	OR	OR [95% CI]		p-value
rs1652507 : rs9458001	2083	590	1.173609812	0.825292983	1.66893457	0.37286	0.563288315	0.427360903	0.699215728	6.93E-16	1.110956	7.78E-01	1.586991	0.5630602
rs140570886	2083	590	1.461838685	0.821001368	2.602885235	0.19707	1.58287126	1.383616228	1.782126291	2.22E-52	1.254036	6.90E-01	2.280472	0.4581362

Note:

Lp(a)*: The original Lp(a) level in mg/dl is highly left-skewed, therefore here raw Lp(a) levels were first inverse normal transformed and then followed with the corresponding analysis.

Supplementary table 10.
Summary statistics for rs140570886 additive effect on CAD risk, total Lp(a) level, apo(a) isoform and Lp(a) levels adjusted on apa(a) isoform

Test item		Odds Ratio (CAD)					Lp(a)*				Lp(a) adjusted for isoform*			apo(a) isoform*		
		N individuals / Haplotype frequencies	OR	OR [95% CI]		p-value	N individuals / Haplotype frequencies	beta	se	p-value	beta	se	p-value	beta	se	p-value
**rs140570886 dosage**		29755	1.9831	1.7235	2.2819	1.14E-21	5953	1.5381	0.0791	9.53E-82	1.1067	0.0686	1.94E-57	-5.7338	0.5932	3.55E-26
Relative effects for rs14 genotypes	T/T	28771	1.0000	1.0000	1.0000	0	5790		1.0000	1		1.0000	1	1.0000	1.0000	1
	T/C	976	1.8787	1.6310	2.1641	2.32E-18	163	1.5381	0.0791	9.53E-82	1.1067	0.0686	1.94E-57	-5.7338	0.5932	3.55E-26
	C/C	8	142181.27	2.97E-77	6.80E+86	0.90	0									

Replication

Test item		Odds Ratio Coronary Artery Diseases					Odds Ratio Aortic Valve Stenosis					Odds Ratio (Perivascular Arterial Diseases)				
		N individuals / Haplotype frequencies	OR	OR [95% CI]		p-value	N individuals / Haplotype frequencies	OR	OR [95% CI]		p-value	N individuals / Haplotype frequencies	OR	OR [95% CI]		p-value
**rs140570886 dosage**	**UKBB**	312312	1.4617	1.3726	1.5566	2.77E-32	344949	1.7132	1.4031	2.0917	1.25E-07	344949	1.4257	1.2204	1.6657	7.83E-06
Relative effects for rs14 genotypes	T/T	302524	1.0000	1.0000	1.0000	0										
	T/C	9717	1.4559	1.3673	1.5503	9.97E-32										
	C/C	71	1.7718	0.9073	3.4599	0.09										

Lp(a)*: The original Lp(a) level in mg/dl is highly left-skewed, therefore here raw Lp(a) levels were first inverse normal transformed and then followed with the corresponding analysis.
The rarest rs140570886 genotype C/C was not present in te KORA studies.

Supplementary table 11

Anova Table reporting likelihood ratio test results for nested model in the model selection procedure

Model	Residuals. Df	Residuals Deviance	Df	Deviance	P-value
CAD ~ covariates	342046	222020.958			
CAD ~ rs140570886 + covariates	342045	221803.9182	1	217.04	4.0005E-49
CAD ~ rs140570886 + rs9458005 + rs1652507 + covariates	342043	221770.3532	2	33.57	5.1456E-08
CAD ~ rs140570886 + rs9458005 * rs1652507 + covariates	342042	221768.032	1	2.32	0.13
CAD ~ rs140570886 * rs9458005 * rs1652507 + covariates	342039	221754.6071	3	13.42	0.004
CAD ~ rs140570886 * rs9458005 * rs1652507 +rs3798220+ covariates	342038	221752.675	1	1.93	0.16
CAD ~ rs140570886 * rs9458005 * rs1652507 * rs3798220+ covariates	342031	221746.45	7	6.22	0.51

Supplementary table 12

Summary statistics for the rs140570886, rs1652507 and rs9458001 and their interactions in the final 3-way interaction model

with 10 MDS component of the genetic variance as covariates

Variable	Estimate	Std. Error	P-value
rs140570886	0.08	0.14	0.55
rs9458001	-0.02	0.01	0.07
rs1652507	-0.07	0.01	3.75E-08
rs140570886:rs9458001	0.29	0.13	0.02
rs140570886:rs1652507	0.25	0.12	0.04
rs9458001:rs1652507	0.00	0.03	0.92
rs140570886:rs9458001:rs1652507	-0.13	0.11	0.23

* Note : Tables showing the results of the same analysis with 3,5 or 7 MDS components are provided in Supplementary Tables 18-20

Supplementary table 13

Summary statistics for the relatives effect of rs1652507-rs9458001-rs140570886 haplotypes on CAD risk

Test item		Odds Ratio (CAD)				
		**N individuals / Haplotype frequencies**	**OR**	**OR [95% CI]**		**P-value**
Relative effects forrs1652507-rs9458001-rs140570886 haplotypes on CAD risk	[TGT]	0.64	1.00	1.00	1.00	0.00
	[TGC]	0.00	1.14	0.22	5.95	0.88
	[TAT]	0.20	0.98	0.94	1.02	0.25
	[TAC]	2.23E-05	0.40	1.11E-10	1468733164	0.94
	[CGT]	0.14	0.89	0.84	0.93	3.58E-06
	[CGC]	0.00	1.92	1.17	3.15	0.01
	[CAT]	0.00	1.25	0.84	1.84	0.27
	[CAC]	0.01	1.89	1.64	2.18	0.00

Note:

Relative effects for haplotypes were estimated with the implementation of R package 'hapassoc'.

The function "pre.hapassoc" was implemented first to generate an input list of possible haplotypes that are compatible with each individual's genotype, and create dummy individuals where haplotypes are uncertain (Burkett et al, 2006).

Then the haplotype association anlayses were conducted based on generalized linear models using the expectation-maximization (EM) algorithm.

It appears that the SE couldn't be reliably computed for the triple minor allele haplotype, and the p-value is therefore to be ignored for this haplotype.

Supplementary table 15

ANOVA Table reporting likelihood ratio test results for nested model in the model selection procedure with 7 MDS components of the genetic variance matrix

Model	Residuals. Df	Residuals De	Df	Deviance	P-value
CAD ~ covariates	342049	222115.38	NA	NA	NA
CAD ~ rs140570886 + covariates	342048	221899.37	1	216.01	6.72E-49
CAD ~ rs140570886 + rs9458005 + rs1652507 + covariates	342046	221866.01	2	33.36	5.71E-08
CAD ~ rs140570886 + rs9458005 * rs1652507 + covariates	342045	221863.77	1	2.24	0.13
CAD ~ rs140570886 * rs9458005 * rs1652507 + covariates	342042	221850.31	3	13.46	0.004
CAD ~ rs140570886 * rs9458005 * rs1652507 +rs3798220+ covariates	342041	221848.19	1	2.12	0.15
CAD ~ rs140570886 * rs9458005 * rs1652507 * rs3798220+ covariates	342034	221841.85	7	6.34	0.5

Supplementary table 16

ANOVA Table reporting likelihood ratio test results for nested model in the model selection procedure with 5 MDS components of the genetic variance matrix

Model	Residuals. D	Residuals De	Df	Deviance	P-value
CAD ~ covariates	342049	222115.38	NA	NA	NA
CAD ~ rs140570886 + covariates	342048	221899.37	1	216.01	6.72E-49
CAD ~ rs140570886 + rs9458005 + rs1652507 + covariates	342046	221866.01	2	33.36	5.71E-08
CAD ~ rs140570886 + rs9458005 * rs1652507 + covariates	342045	221863.77	1	2.24	0.13
CAD ~ rs140570886 * rs9458005 * rs1652507 + covariates	342042	221850.31	3	13.46	0.004
CAD ~ rs140570886 * rs9458005 * rs1652507 +rs3798220+ covariates	342041	221848.19	1	2.12	0.15
CAD ~ rs140570886 * rs9458005 * rs1652507 * rs3798220+ covariates	342034	221841.85	7	6.34	0.5

Supplementary table 17

ANOVA Table reporting likelihood ratio test results for nested model in the model selection procedure with 3 MDS components of the genetic variance matrix

Model	Residuals. Df	Residuals Deviance	Df	Deviance	P-value
**CAD ~ covariates**	342053	222219.8	NA	NA	NA
**CAD ~ rs140570886 + covariates**	342052	222002.8	1	217.09	3.90E-49
**CAD ~ rs140570886 + rs9458005 + rs1652507 + covariates**	342050	221970.2	2	32.58	8.42E-08
**CAD ~ rs140570886 + rs9458005 * rs1652507 + covariates**	342049	221967.9	1	2.3	0.13
**CAD ~ rs140570886 * rs9458005 * rs1652507 + covariates**	342046	221954.7	3	13.15	0.004
**CAD ~ rs140570886 * rs9458005 * rs1652507 +rs3798220+ covariates**	342045	221953	1	1.76	0.19
**CAD ~ rs140570886 * rs9458005 * rs1652507 * rs3798220+ covariates**	342038	221946.7	7	6.23	0.51

Supplementary table 18

Summary statistics for the rs140570886, rs1652507 and rs9458001 and their interactions in the final 3-way interaction model

with 7 MDS component of the genetic variance as covariates

Variable	Estimate	Std. Error	P-value
rs140570886	0.077	0.135	0.567
rs9458001	-0.02	0.011	0.074
rs1652507	-0.074	0.013	4.355E-08
rs140570886:rs9458001	0.291	0.126	0.021
rs140570886:rs1652507	0.251	0.12	0.0357
rs9458001:rs1652507	0.002	0.026	0.9351
rs140570886:rs9458001:rs1652507	-0.137	0.11	0.214

Supplementary table 19

Summary statistics for the rs140570886, rs1652507 and rs9458001 and their interactions in the final 3-way interaction model

with 5 MDS component of the genetic variance as covariates

Variable	Estimate	Std. Error	P-value
rs140570886	0.076	0.135	0.304
rs9458001	-0.02	0.011	0.574
rs1652507	-0.074	0.013	0.074
rs140570886:rs9458001	0.291	0.126	0.021
rs140570886:rs1652507	0.252	0.12	0.036
rs9458001:rs1652507	0.002	0.026	0.936
rs140570886:rs9458001:rs1652507	-0.137	0.11	0.213

Supplementary table 20

Summary statistics for the rs140570886, rs1652507 and rs9458001 and their interactions in the final 3-way interaction model

with 3 MDS component of the genetic variance as covariates

Variable	Estimate	Std. Error	P-value
rs140570886	0.082	0.135	0.546
rs9458001	-0.019	0.011	0.084
rs1652507	-0.073	0.013	5.72E-08
rs140570886:rs9458001	0.288	0.126	0.022
rs140570886:rs1652507	0.247	0.12	0.039
rs9458001:rs1652507	0.003	0.026	0.911
rs140570886:rs9458001:rs1652507	-0.135	0.11	0.222
